# Perioperative NT-proBNP in Lung Cancer Surgery: A Narrative Review of Mechanisms, Predictive Value, and Clinical Implications

**DOI:** 10.3390/jcm15176606

**Published:** 2026-08-26

**Authors:** Mădălina Butaș, Sonia Elena Popovici, Stelian Adrian Ritiu, Gabriel Veniamin Cozma, Vasile Gaborean, Iulia Najette Crintea, Maria Sala-Cirtog, Alina Ramona Buzatu, Roxana Buzas, Marilena Dinuți

**Affiliations:** 1 Faculty of Medicine, Victor Babes University of Medicine and Pharmacy Timisoara, 300041 Timisoara, Romania; madalina.moise@umft.ro (M.B.); mariasala.cirtog@umft.ro (M.S.-C.); buzatu.ramona@umft.ro (A.R.B.); dinuti.marilena@umft.ro (M.D.); 2 Emergency Municipal Clinical Hospital Timisoara, 300079 Timisoara, Romania; 3 Doctoral School, Victor Babes University of Medicine and Pharmacy Timisoara, 300041 Timisoara, Romania; 4 Anaesthesia and Intensive Care Research Center (CCATITM), Victor Babes University of Medicine and Pharmacy Timisoara, 300041 Timisoara, Romania; 5 Discipline of Surgical Semiology I and Thoracic Surgery, Department of Surgery I, Victor Babes University of Medicine and Pharmacy Timisoara, 300041 Timisoara, Romania; 6 Thoracic Surgery Research Center, Victor Babes University of Medicine and Pharmacy Timisoara, 300041 Timisoara, Romania; 7 Department of Surgery I, Faculty of Medicine, Victor Babes University of Medicine and Pharmacy, 300041 Timisoara, Romania; 8 Discipline of Biochemistry, Department of Biochemistry and Pharmacology, Faculty of Medicine, Victor Babes University of Medicine and Pharmacy Timisoara, 300041 Timisoara, Romania; 9 1st Medical Semiology, Internal Medicine, Department V, Victor Babes University of Medicine and Pharmacy Timisoara, 300041 Timisoara, Romania; buzas.dana@umft.ro; 10 Center for Advanced Research in Cardiovascular Pathology and in Hemostaseology, 300041 Timisoara, Romania

**Keywords:** NT-proBNP, BNP, lung cancer surgery, pulmonary resection, perioperative risk stratification, postoperative atrial fibrillation, right ventricular dysfunction, one-lung ventilation, perioperative myocardial injury

## Abstract

**Background**: NT-proBNP is a mechanistically grounded biomarker of ventricular wall stress with established prognostic value in cardiac surgery, but its perioperative role in lung cancer resection remains incompletely characterised. Pulmonary resection imposes unique haemodynamic stressors—including one-lung ventilation-induced right ventricular afterload increase, permanent pulmonary vascular bed reduction, and ischaemia–reperfusion injury—that create a distinct biological context for natriuretic peptide elevation not represented in general surgical cohorts. **Methods**: A narrative review of the literature was conducted through systematic searches of PubMed, EMBASE/MEDLINE, and the Cochrane Library (January 2000–June 2026), supplemented by manual reference screening. Approximately 135 articles were included in the final synthesis. **Results**: Perioperative NT-proBNP elevation predicts postoperative atrial fibrillation, major adverse cardiovascular events, and long-term survival after lung resection, with effect sizes substantially exceeding those reported in general surgical populations. The PRESAGE trial established that NT-proBNP-guided prophylaxis reduces postoperative atrial fibrillation from 40% to 6% in high-risk patients. No thoracic surgery-specific NT-proBNP threshold has been prospectively validated in a multicentre setting, and approximately half the evidence base derives from BNP rather than NT-proBNP assays, precluding direct threshold synthesis. Surgical approach—particularly robot-assisted thoracoscopy—modulates postoperative biomarker elevation through OLV duration rather than inflammatory burden alone. The combination of NT-proBNP with high-sensitivity troponin identifies a dual-elevation subgroup with a MACE rate of 18.4%. **Conclusions**: NT-proBNP measurement is clinically actionable in thoracic surgery but requires assay-standardised, multicentre validation of population-specific thresholds stratified by resection extent and surgical approach. A dual biomarker strategy combining NT-proBNP with high-sensitivity troponin represents the most evidence-based perioperative risk stratification framework currently available.

## 1. Introduction

Lung cancer remains the leading cause of cancer-related mortality worldwide, accounting for approximately 1.8 million deaths annually [1]. Surgical resection represents the cornerstone of curative-intent treatment for early-stage non-small-cell lung cancer, with five-year survival rates of 60–90% for stage I disease following complete resection [2]. As surgical candidacy expands to older and more comorbid populations—driven by advances in minimally invasive technique, improved anaesthetic management, and increasingly sensitive staging—the accurate preoperative identification of patients at elevated cardiovascular risk has become one of the central challenges in thoracic oncology. Postoperative cardiovascular complications, most prominently atrial fibrillation, perioperative myocardial injury, and right ventricular dysfunction, occur in 10–20% of patients undergoing pulmonary resection and contribute disproportionately to postoperative morbidity, prolonged hospital stay, and long-term survival impairment independent of oncological outcome [3,4].

Pulmonary resection imposes a haemodynamic burden that is qualitatively distinct from other major surgical procedures. One-lung ventilation, required for operative exposure in virtually all pulmonary resections, acutely increases right ventricular afterload through hypoxic pulmonary vasoconstriction and single-lung positive-pressure ventilation [5,6]. Permanent reduction in the pulmonary vascular bed following resection creates a proximal wave reflection site in the operative pulmonary artery, driving sustained right ventricular–pulmonary artery uncoupling that persists for months beyond the index procedure [7,8]. These mechanisms—layered upon the systemic inflammatory response to surgical trauma and ischaemia–reperfusion injury—generate a perioperative haemodynamic stress profile for which standard cardiac risk scores such as the Revised Cardiac Risk Index were not designed and in which their predictive accuracy is correspondingly limited [2,9].

NT-proBNP, the inert N-terminal cleavage product of proBNP released in response to ventricular wall stretch, has emerged as a promising perioperative biomarker in this context [10]. Beyond its established role in acute cardiovascular presentations, interest is growing in the use of cardiac biomarkers—including NT-proBNP, high-sensitivity troponin, and D-dimer—measured outside the context of acute cardiovascular illness, to characterise subclinical cardiovascular dysfunction in otherwise asymptomatic populations, with recent evidence linking such biomarkers to structural and functional cardiac parameters, subclinical atherosclerosis, and arterial stiffness [11]. Its long half-life of approximately 120 min, stability at room temperature, and independence from neprilysin inhibition make it technically well suited to perioperative monitoring, and it has demonstrated superior predictive performance compared to BNP in the thoracic surgery setting [12]. At the clinical level, landmark studies including the PRESAGE trial have demonstrated that preoperative NT-proBNP elevation identifies a subgroup of lung cancer surgery patients in whom targeted pharmacological prophylaxis can reduce postoperative atrial fibrillation from 40% to 6%—an effect size that places NT-proBNP among the most clinically actionable biomarkers in perioperative medicine [3]. More recently, the combination of NT-proBNP with high-sensitivity cardiac troponin I has been shown to identify a dual-elevation subgroup with a major adverse cardiovascular event rate of 18.4%, substantially exceeding the risk captured by either biomarker alone [13].

Despite this evidence, important controversies remain. No validated, assay-specific NT-proBNP threshold exists for thoracic surgery populations, and approximately half the published evidence derives from BNP rather than NT-proBNP assays, creating a bifurcated literature that resists direct synthesis [14,15]. The emerging influence of surgical approach—particularly the paradoxical finding that robot-assisted thoracoscopy is associated with greater perioperative myocardial injury than video-assisted thoracoscopy despite lower systemic inflammation, likely through longer one-lung ventilation duration—challenges existing threshold assumptions derived predominantly from open thoracotomy cohorts [4]. Furthermore, the optimal timing, frequency, and clinical integration of perioperative NT-proBNP measurement remain undefined, and the long-term haemodynamic consequences of pulmonary vascular bed reduction—including progressive pulmonary hypertension and right ventricular remodelling—suggest that the prognostic horizon of perioperative biomarker measurement extends well beyond the conventional perioperative window [7,16].

This narrative review synthesises the current evidence on the perioperative role of NT-proBNP in lung cancer surgery, encompassing its biological basis, the pathophysiological mechanisms linking pulmonary resection to natriuretic peptide elevation, its predictive value across the spectrum of perioperative outcomes, its relationship to other perioperative biomarkers, and the emerging influence of surgical approach on biomarker dynamics. We further identify the critical evidence gaps that constrain clinical implementation, propose a pragmatic four-tier framework for perioperative NT-proBNP interpretation, and outline the multicentre prospective research agenda required to translate this biomarker into routine thoracic surgical care.

## 2. Materials and Methods

A narrative review of the literature was conducted through a systematic search of PubMed, EMBASE/MEDLINE, and the Cochrane Library, covering publications from January 2000 to June 2026. The following search terms were used in combination: “NT-proBNP”, “BNP”, “natriuretic peptide”, “B-type natriuretic peptide”, “lung resection”, “pulmonary resection”, “lung cancer surgery”, “thoracic surgery”, “one-lung ventilation”, “perioperative”, “postoperative atrial fibrillation”, “major adverse cardiovascular events”, “right ventricular function”, “right ventricular dysfunction”, “pulmonary vascular resistance”, and “perioperative myocardial injury”. Results were limited to publications in the English language. The search was supplemented by manual screening of reference lists of included articles to identify additional relevant sources. Studies were considered for inclusion if they reported original data, systematic analyses, or meta-analyses on natriuretic peptide measurement in the perioperative context of lung resection or thoracic surgery. Guideline documents from the American Heart Association/American College of Cardiology (AHA/ACC), European Society of Cardiology (ESC), and the European Respiratory Society/European Society of Thoracic Surgeons (ERS/ESTS) were additionally reviewed for relevant recommendations. Case reports, editorials, letters, and studies not specific to thoracic or cardiothoracic surgery populations were excluded. Following deduplication and screening, approximately 135 articles were included in the final narrative synthesis.

**Figure 1:**
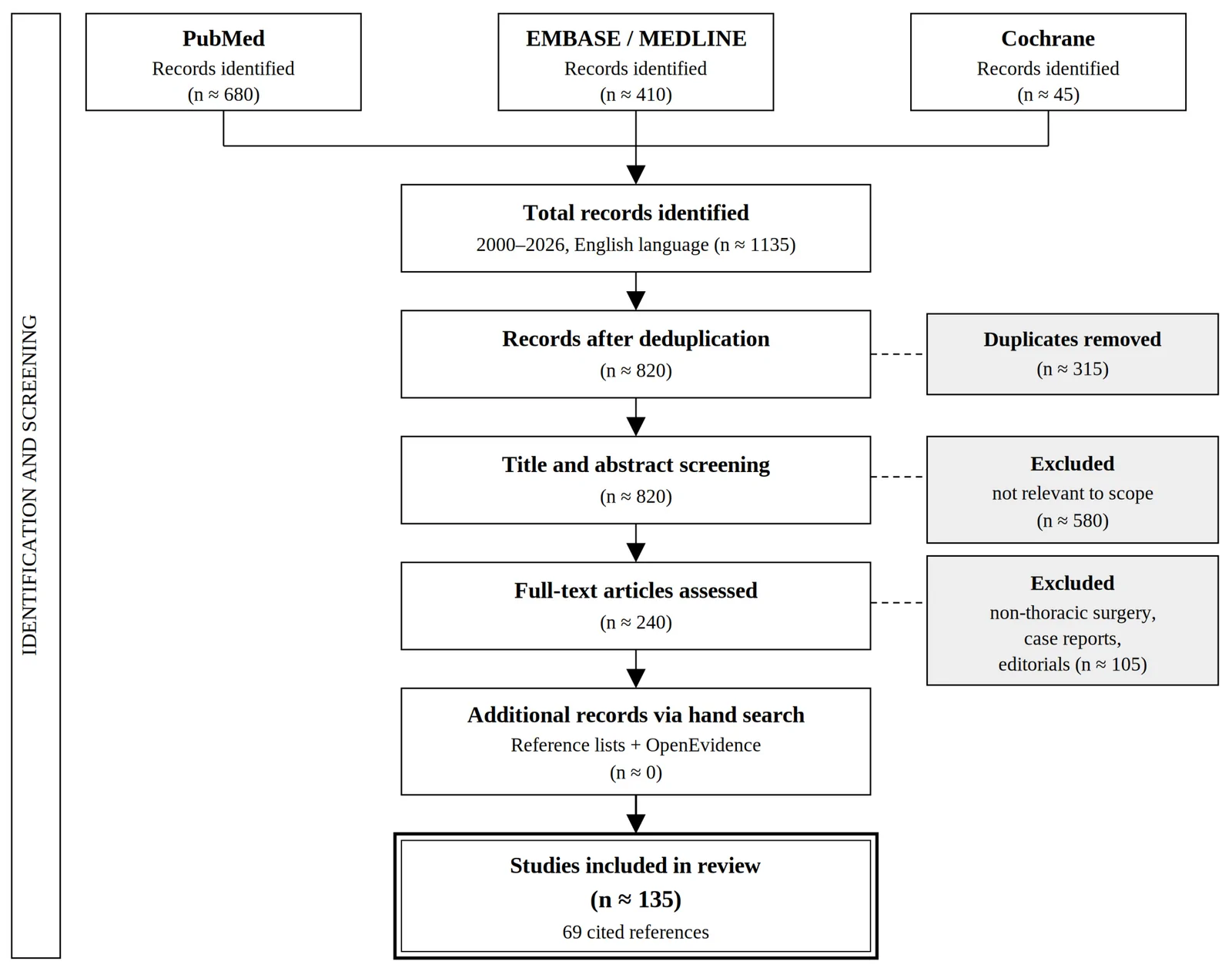
Literature search and selection flow diagram. Records were identified through systematic searches of PubMed, EMBASE/MEDLINE, and the Cochrane Library (January 2000–June 2026).

To enhance the transparency of the evidence synthesis, included studies were qualitatively appraised according to design (randomised controlled trial, prospective cohort, retrospective cohort, or preclinical/mechanistic study), sample size, and outcome ascertainment method; this hierarchy is made explicit throughout the narrative synthesis by identifying study design and cohort size alongside each reported finding. Given the heterogeneity of study types included in this narrative review—spanning randomised trials, prospective and retrospective observational cohorts, and preclinical models—a formal risk-of-bias instrument such as the Newcastle-Ottawa Scale was not applied systematically across the full evidence base, as such tools are designed for comparable observational study designs rather than the mixed clinical and mechanistic literature synthesised here. This represents a limitation of the present narrative approach, and future systematic reviews or meta-analyses restricted to comparable observational cohorts would benefit from formal quality scoring to further strengthen methodological transparency (Figure 1).

## 3. NT-proBNP Biology and Physiology

**Figure 2:**
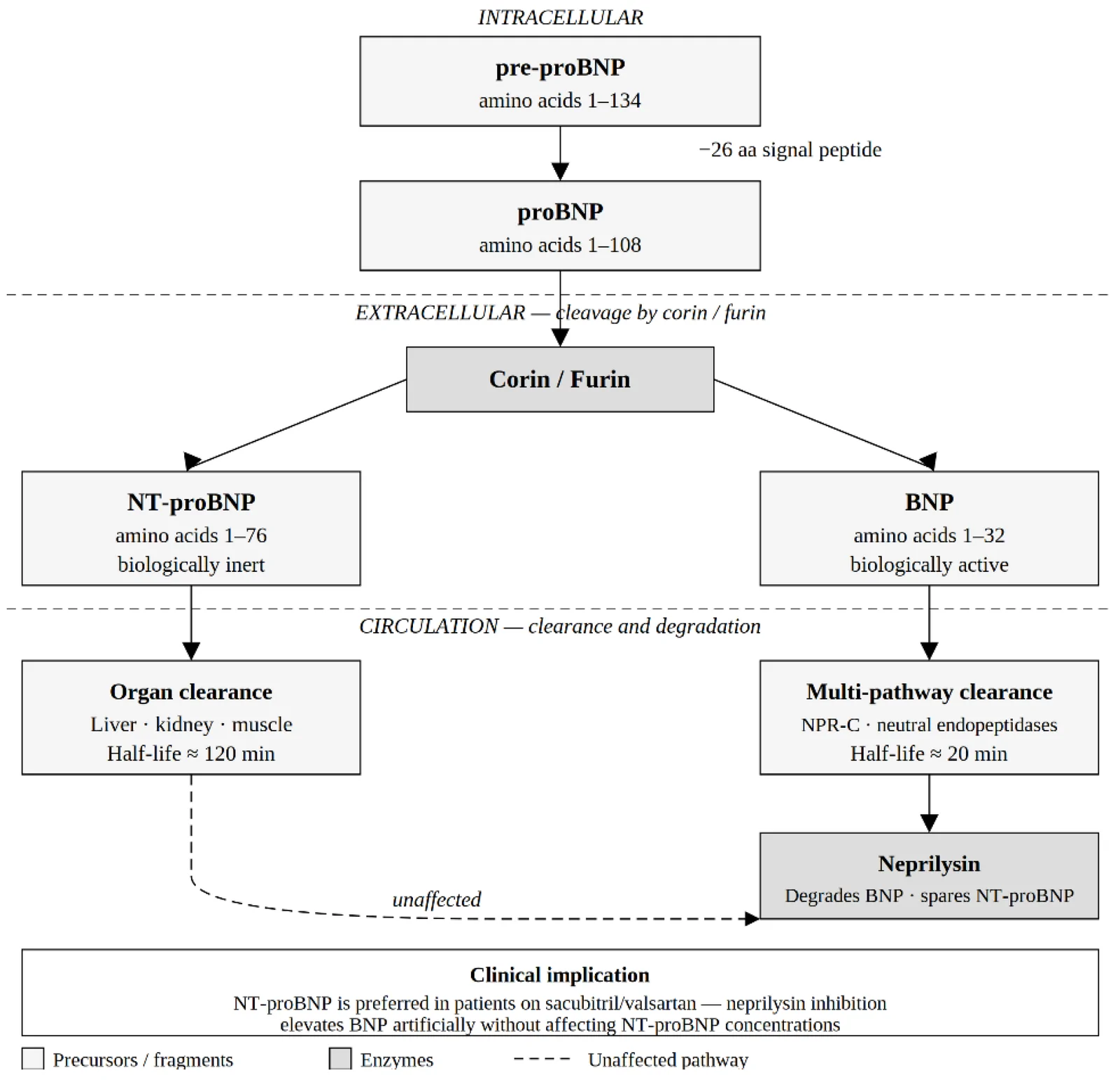
Schematic of the natriuretic peptide biosynthetic cascade and clearance pathways. Pre-proBNP undergoes signal peptide cleavage to form proBNP, which is subsequently processed by corin and furin into biologically active BNP (1–32) and the inert fragment NT-proBNP (1–76). The two fragments differ markedly in their clearance mechanisms and half-lives. NT-proBNP is unaffected by neprilysin, making it the preferred assay in patients receiving sacubitril/valsartan therapy.

B-type natriuretic peptide (BNP) and its N-terminal fragment (NT-proBNP) are synthesised in ventricular cardiomyocytes in response to increased wall stress, volume overload, and ischaemia. The biosynthetic cascade, illustrated in Figure 2, begins with pre-proBNP (1–134), which undergoes cleavage of its 26-amino-acid signal peptide to yield proBNP (1–108). This precursor is subsequently processed by the serine proteases furin and corin into two distinct fragments: the biologically active BNP (1–32), which exerts natriuretic, vasodilatory, and antifibrotic effects, and the haemodynamically inert NT-proBNP (1–76) [16].

Although BNP and NT-proBNP are released in equimolar quantities, their pharmacokinetic profiles differ substantially. BNP is cleared via multiple pathways—natriuretic peptide receptor-C (NPR-C), neutral endopeptidases, and passive renal excretion—resulting in a relatively short half-life of approximately 20 min. NT-proBNP, by contrast, is eliminated primarily by high-flow organs including the liver, skeletal muscle, and kidneys, conferring a markedly longer half-life of approximately 120 min [17]. This prolonged stability renders NT-proBNP less susceptible to acute fluctuations and more suitable for serial perioperative monitoring. An additional practical advantage is its resistance to degradation by neprilysin, making it the preferred biomarker in patients receiving sacubitril/valsartan therapy, as neprilysin inhibition artificially elevates BNP levels without affecting NT-proBNP concentrations [18].

Several physiological and pathological variables are known to influence circulating NT-proBNP levels independently of cardiac function, and their recognition is essential when interpreting perioperative values. Age exerts a pronounced effect: median NT-proBNP concentrations rise from approximately 21 pg/mL in males under 30 years of age to 281 pg/mL in those aged 80 years or older. Sex also contributes significantly, with women demonstrating consistently higher levels than men, a difference attributed to androgen-mediated suppression of BNP gene expression in males [18,19]. These age- and sex-specific reference ranges must therefore be applied when defining perioperative thresholds in thoracic surgery populations, which tend to be older and predominantly male.

Renal function represents another critical confounding variable. A robust log-linear relationship exists between declining estimated glomerular filtration rate (eGFR) and rising NT-proBNP concentrations, with this association being stronger for NT-proBNP than for BNP—likely reflecting the greater dependence of NT-proBNP on renal clearance [20,21]. While mild renal dysfunction may exert only a modest effect after correction for age and sex, moderate-to-severe impairment can substantially inflate NT-proBNP values independent of cardiac status [20,21]. Conversely, obesity is paradoxically associated with lower circulating NT-proBNP concentrations, despite representing one of the strongest drivers of metabolic syndrome and cardiovascular risk—a relationship that remains incompletely understood. The originally proposed explanation attributed this effect to increased expression of the natriuretic peptide clearance receptor (NPR-C) in adipose tissue, which enhances receptor-mediated internalisation and degradation of circulating natriuretic peptides. However, this mechanism alone cannot fully account for the phenomenon: NT-proBNP, unlike biologically active BNP, is not cleared via NPR-C or degraded by neprilysin, yet it demonstrates an inverse relationship with body mass index comparable in magnitude to that of BNP. This observation has shifted mechanistic attention toward impaired myocardial natriuretic peptide production and secretion in obesity, potentially mediated by insulin resistance, adipokine-driven inhibition of natriuretic peptide gene transcription, and reduced corin-dependent proBNP processing, rather than by peripheral clearance alone. The clinical consequence of this paradox is clinically significant: because obesity simultaneously increases cardiovascular risk and lowers natriuretic peptide concentrations, standard NT-proBNP thresholds may under-detect subclinical cardiac stress and heart failure with preserved ejection fraction in obese patients, supporting the use of lower, weight-adjusted diagnostic cut-offs in this population [22]. This phenomenon has clinical relevance in the perioperative setting, as at least 30% of patients with heart failure with preserved ejection fraction (HFpEF) and elevated filling pressures may present with NT-proBNP values below standard diagnostic thresholds, particularly those who are younger and more obese [18,23]. Finally, atrial fibrillation—a complication of particular relevance in thoracic surgery, where its postoperative incidence reaches 10–20%—independently elevates NT-proBNP levels, further complicating the interpretation of perioperative measurements in this population [20].

## 4. Pathophysiological Mechanisms Linking Thoracic Surgery to NT-proBNP Elevation

The primary trigger for BNP and NT-proBNP release is myocardial wall stretch rather than transmural pressure per se. In the setting of volume expansion or pressure overload, increased wall stress initiates de novo transcription of pre-proBNP in ventricular myocytes, which undergoes cleavage to proBNP (1–108) and subsequently to biologically active BNP (1–32) and the inert fragment NT-proBNP (1–76) [10,24]. Unlike atrial natriuretic peptide (ANP), which is stored in atrial secretory granules and released rapidly in response to even minor stimuli such as exercise, BNP has minimal granule storage and its release instead reflects gene transcription regulated by acute haemodynamic stress [10]. This transcriptional basis has been directly demonstrated in cultured neonatal rat ventricular cardiomyocytes, where BNP mRNA increases to near-maximal levels within one hour of hypertrophic stimulation—kinetics comparable to the immediate-early gene c-fos—and where transcriptional blockade with actinomycin D completely abolishes the response, confirming BNP’s role as a rapidly inducible “emergency” cardiac hormone against ventricular overload [25]. This burst-synthesis mechanism has a direct clinical correlate in thoracic surgery: the characteristic 12–24 h delay between the onset of cardiac stress and peak NT-proBNP elevation observed after lung resection is a consequence of the time required for de novo gene expression, messenger RNA transcription, and peptide processing—not simply a reflection of biomarker clearance kinetics [26,27].

### 4.1. Intraoperative Right Ventricular Afterload Increase During One-Lung Ventilation

One-lung ventilation (OLV) creates a unique and complex haemodynamic environment through several converging mechanisms. When the nondependent lung is collapsed, alveolar hypoxia triggers hypoxic pulmonary vasoconstriction (HPV) in the nondependent pulmonary vasculature, diverting blood flow to the ventilated dependent lung. This response is mediated by a redox-based oxygen sensor within pulmonary artery smooth muscle cells: hypoxia inhibits voltage-gated potassium channels, causing membrane depolarisation and activation of L-type calcium channels, which drives vasoconstriction. While HPV reduces intrapulmonary shunt from a theoretical 50% to approximately 25–31% of cardiac output, it simultaneously increases total pulmonary vascular resistance (PVR). In a landmark human study, Carlsson et al. demonstrated that unilateral hypoxia during anaesthesia increased PVR of the hypoxic lung by 54% and mean pulmonary artery pressure (PAP) by 14% within 15 min, with both parameters continuing to rise for up to 60 min [5,28,29]. This acute right ventricular pressure stimulus finds a direct preclinical correlate in a rat model of pulmonary arterial banding, in which right ventricular-specific induction of ANP and BNP mRNA was detected within a single day of pressure overload onset, while left ventricular expression—unaffected by the intervention—remained unchanged, confirming that natriuretic peptide induction under isolated right ventricular afterload increase is a direct, chamber-specific transcriptional response rather than a systemic phenomenon [30].

Mechanical effects of single-lung positive-pressure ventilation further compound this afterload increase. During OLV, the entire tidal volume is delivered to one lung, creating supraphysiologic parenchymal strain. The impact of positive end-expiratory pressure (PEEP) on right ventricular (RV) function in this context is clinically significant: Kim et al. demonstrated in 36 lung cancer patients undergoing OLV that a PEEP of 10 cmH_2_O significantly decreased RV fractional area change from 46 ± 10% at baseline to 41 ± 9% (*p* = 0.001) and increased the RV myocardial performance index from 0.52 ± 0.12 to 0.64 ± 0.21 (*p* < 0.001) compared to lower PEEP settings [31,32]. Both hyperinflation and hypoinflation of the dependent lung increase RV afterload through compression of juxta-alveolar capillaries, albeit via different mechanisms. Importantly, ventilatory mode modulates this response: Al Shehri et al. showed that pressure-controlled ventilation during OLV resulted in significantly better RV systolic function—reflected by a systolic tricuspid annular velocity of 10.1 versus 5.8 cm/s (*p* < 0.001)—and lower RV end-systolic volumes compared to volume-controlled ventilation, suggesting that the choice of ventilatory strategy directly influences the magnitude of natriuretic peptide release through its effects on RV wall stress [33].

The net haemodynamic consequence of OLV varies with the patient’s underlying RV reserve. While Brock et al. found that OLV alone without CO_2_ insufflation did not significantly alter cardiac index in a video-assisted thoracoscopic surgery (VATS) setting—likely reflecting compensatory mechanisms in patients with preserved RV function—patients with pre-existing RV compromise or pulmonary hypertension are at risk for clinically significant RV afterload mismatch, representing the precise haemodynamic stimulus for ventricular NT-proBNP release [31,34,35].

### 4.2. Acute Pulmonary Vascular Bed Reduction Following Resection

The permanent loss of pulmonary vascular bed created by lung resection establishes a distinct and sustained stimulus for NT-proBNP elevation that persists well beyond the intraoperative period. Using cardiac magnetic resonance imaging with wave intensity analysis, Glass et al. demonstrated that lobectomy creates a proximal reflection site in the operative pulmonary artery, with wave reflection increasing from 4.3% preoperatively to 9.5% on postoperative day 2 (*p* < 0.001) and remaining elevated at 8.0% at two months. This increased pulsatile afterload—mechanistically distinct from mean PVR—was directly correlated with impaired RV ejection fraction (r = −0.480, *p* = 0.028) and blood flow redistribution toward the nonoperative lung (r = −0.545, *p* = 0.011), highlighting the importance of pulsatile haemodynamics as a driver of postoperative RV stress [8].

McCall et al. provided the first cardiac MRI evidence that RV ejection fraction deteriorates from 50.5% preoperatively to 45.6% on postoperative day 2 and remains depressed at 44.9% at two months (*p* = 0.003), while left ventricular ejection fraction was unchanged (*p* = 0.621) [20]. The deterioration in the stroke volume-to-end-systolic volume ratio—from 1.0 to 0.8 (*p* = 0.011)—indicates a mismatch between RV contractility and afterload, representing the hallmark of RV–pulmonary artery (RV-PA) uncoupling [7].

A dose–response relationship between resection extent and natriuretic peptide elevation has been established at both the haemodynamic and biochemical level. Tayama et al. directly measured natriuretic peptides and pulmonary haemodynamics after lung resection and demonstrated that pneumonectomy patients exhibited significantly higher ANP and BNP concentrations than lobectomy patients on postoperative days 3 and 7, with plasma BNP in the pneumonectomy group directly correlating with total PVR on postoperative day 3—establishing a mechanistic link between vascular bed reduction, RV afterload, and natriuretic peptide release [26].

Echocardiographic data reinforce this dose–response pattern: Amar et al. showed that pneumonectomy but not lobectomy was associated with postoperative pulmonary hypertension, with particularly pronounced effects in patients with FEV_1_ below 60% predicted [36]. Foroulis et al. confirmed that at six months, mean pulmonary artery systolic pressure (PASP) after pneumonectomy was significantly higher than after lobectomy (40.5 vs. 32.9 mmHg, *p* = 0.012), with right pneumonectomy producing the highest pressures (48.3 vs. 35.3 mmHg for left pneumonectomy, *p* = 0.002), attributable to the greater magnitude of vascular bed loss on the right side [37]. Speckle-tracking echocardiography data from Wang et al. further confirm that both RV and LV longitudinal strain decrease after lung resection in both groups, but with a significantly greater reduction after pneumonectomy (*p* < 0.05), demonstrating the dose-dependent impact of resection extent on biventricular function [38].

### 4.3. Surgical Trauma, Inflammatory Mediators, and Neurohormonal Activation

Beyond pure haemodynamic mechanisms, several additional pathways contribute to perioperative NT-proBNP elevation. Re-expansion of the collapsed lung at the conclusion of OLV induces ischaemia–reperfusion injury of duration-dependent severity, with release of inflammatory cytokines—including IL-6, IL-8, and TNF-α—capable of promoting both local pulmonary injury and systemic myocardial inflammation [39,40]. Endothelial glycocalyx degradation during OLV may represent a common pathway for these effects, as glycocalyx injury increases vascular permeability and is associated with myocardial oedema, both of which constitute potential stimuli for natriuretic peptide synthesis [39,40]. Simultaneously, the ventilated dependent lung experiences hyperperfusion—receiving the entirety of cardiac output during OLV—generating capillary shear stress that, combined with hyperoxia-related oxidative stress, damages the pulmonary endothelium and further increases RV afterload through microvascular dysfunction.

At the neurohormonal level, thoracic surgery activates the renin–angiotensin–aldosterone system through surgical stress, pain, blood loss, and mediastinal manipulation. Angiotensin II and endothelin-1 can augment BNP gene transcription independent of haemodynamic effects, driving natriuretic peptide release as a counter-regulatory response [24,41]. Concurrent volume shifts and sympathetic activation increase both venous return—stimulating ANP through atrial stretch—and ventricular wall stress, providing a simultaneous stimulus for BNP/NT-proBNP release [24,42].

### 4.4. Chronic Pulmonary Vascular Remodeling (Late NT-proBNP Elevation)

The haemodynamic consequences of lung resection are not confined to the acute perioperative period. Sentenac et al. demonstrated in a preclinical model that pneumonectomy leads to progressive pulmonary hypertension—with mean PAP rising to 35 mmHg by postoperative day 28 versus 18 mmHg in sham-operated controls (*p* = 0.001)—driven by muscularization of distal pulmonary arteries (83% vs. 5% muscularized, *p* < 0.001), a process mediated by shear stress-induced paracrine signalling from pulmonary endothelial cells promoting smooth muscle cell proliferation [43]. The long-term clinical implications of this remodelling are significant: Tan et al. demonstrated that RV-PA uncoupling—defined as a TAPSE/PASP ratio below 0.67 mm/mmHg—after lung resection for non-small-cell lung cancer (NSCLC) was associated with substantially reduced survival at both three years (40% vs. 73%, *p* < 0.001) and five years (37% vs. 64%, *p* < 0.001), with diabetes identified as an independent risk factor for postoperative RV dysfunction [16]. At the functional level, Young et al. showed that preoperative BNP predicted functional deterioration—defined as a decreased six-minute walk distance or increased dyspnoea score—at two months after lung resection, with an AUC of 0.82 (*p* = 0.01), suggesting that natriuretic peptide levels identify patients whose RV reserve is insufficient to compensate for the afterload imposed by resection [44].

### 4.5. An Integrated Temporal Model

**Figure 3:**
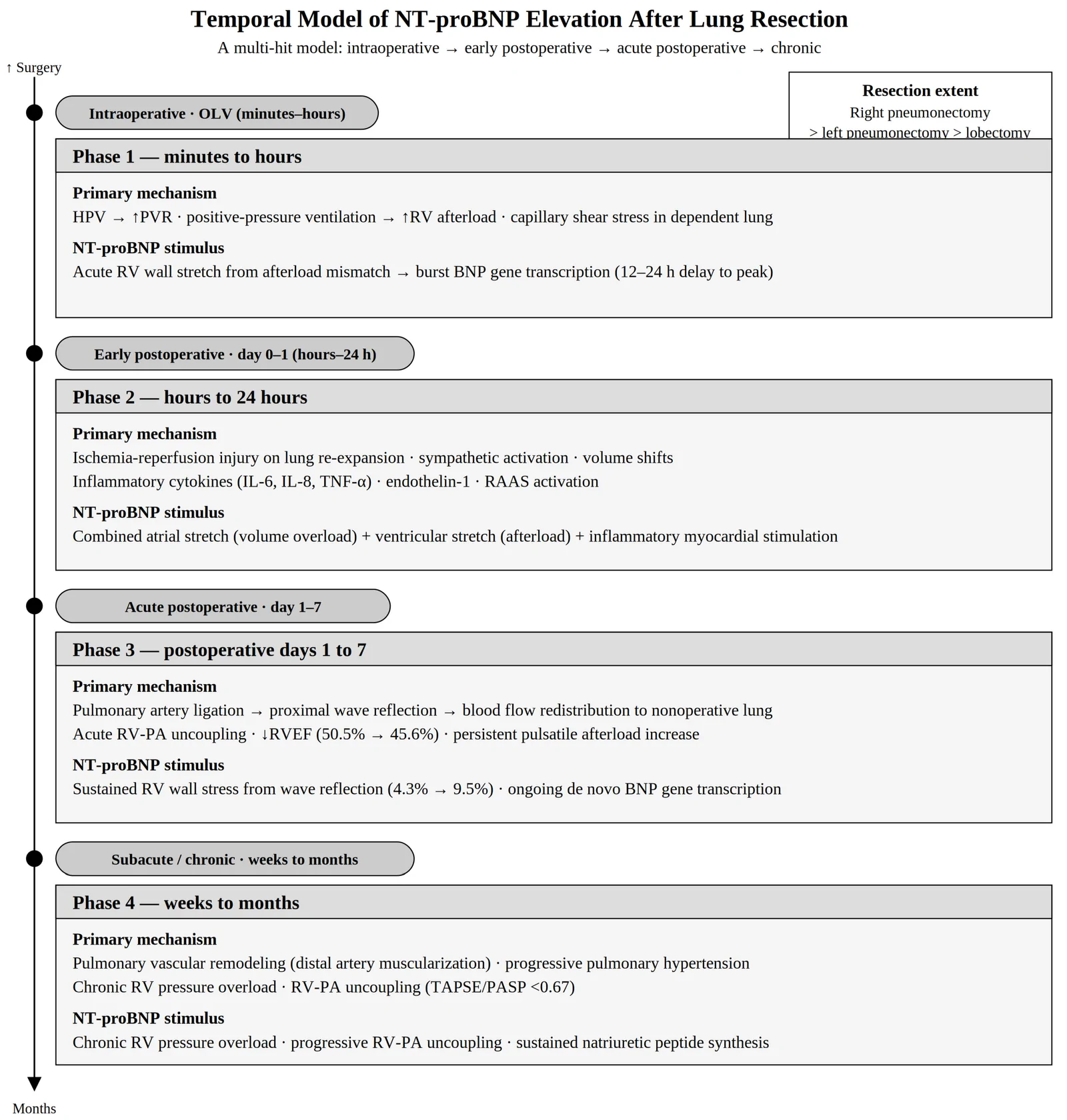
Temporal model of NT-proBNP elevation following lung resection—a multi-hit pathophysiological framework. Four sequential phases are delineated by their predominant mechanism and NT-proBNP stimulus: intraoperative, early postoperative, acute postoperative, and chronic. The magnitude of NT-proBNP elevation follows a dose–response relationship with resection extent across all phases. Phase 1 mechanisms are based on [9,10,12,13,14,15,16,17,18]; Phase 2 mechanisms are based on [24,25,26,27]; Phase 3 mechanisms are based on [19,20,21,22,23]; and Phase 4 mechanisms are based on [28,29,30]. HPV, hypoxic pulmonary vasoconstriction; OLV, one-lung ventilation; PVR, pulmonary vascular resistance; RAAS, renin–angiotensin–aldosterone system; RVEF, right ventricular ejection fraction; RV-PA, right ventricular–pulmonary artery; TAPSE, tricuspid annular plane systolic excursion.

Taken together, these mechanisms suggest a multi-hit model of NT-proBNP elevation in thoracic surgery, unfolding across four temporal phases that are presented in Figure 3. During OLV (minutes to hours), HPV and single-lung positive-pressure ventilation combine to acutely increase RV afterload, producing wall stretch-mediated burst transcription of BNP. In the early postoperative period (hours to 24 h), ischaemia–reperfusion injury on lung re-expansion, sympathetic activation, inflammatory cytokine release, and volume shifts provide a second, overlapping stimulus involving both atrial and ventricular stretch. Between postoperative days 1 and 7, pulmonary artery ligation creates a proximal wave reflection site, redistributing blood flow and sustaining RV-PA uncoupling as the dominant mechanism. Finally, over weeks to months, progressive muscularization of the residual pulmonary vasculature drives pulmonary hypertension and chronic RV pressure overload, maintaining NT-proBNP elevation beyond the period traditionally regarded as perioperative. The dose–response relationship observed across all phases—greatest after right pneumonectomy, intermediate after left pneumonectomy, and least after lobectomy—is mechanistically explained by the proportional increase in PVR, wave reflection magnitude, and RV afterload associated with each resection extent.

### 4.6. Summary of Key Pathophysiological Concepts

NT-proBNP elevation after thoracic surgery is best understood as a multi-hit phenomenon rather than a single haemodynamic event. Intraoperative HPV and positive-pressure ventilation constitute the first hit, producing acute RV afterload mismatch during OLV. Ischaemia–reperfusion injury on lung re-expansion, together with systemic inflammatory and neurohormonal activation, provides a second, overlapping stimulus in the early postoperative hours. Permanent pulmonary vascular bed reduction—with its attendant proximal wave reflection, blood flow redistribution, and sustained RV-PA uncoupling—constitutes the third and most durable hit, one that persists well beyond what is conventionally regarded as the perioperative window. The dose–response relationship between resection extent and NT-proBNP elevation is mechanistically explained at each phase by the proportional increase in PVR, wave reflection magnitude, and RV afterload: greatest after right pneumonectomy, intermediate after left pneumonectomy, and least after lobectomy [26,36,37]. The persistence of RV dysfunction at two months documented by cardiac MRI (McCall et al.) and the progressive muscularization of distal pulmonary arteries demonstrated by Sentenac et al. together provide a pathophysiological rationale for extending NT-proBNP monitoring beyond hospital discharge [7,43]. An important conceptual distinction concerns the complementary roles of NT-proBNP and high-sensitivity troponin in this setting. Troponin elevation after lung resection may reflect mechanisms other than myocardial ischaemia—including RV strain, demand ischaemia from perioperative tachycardia, and direct inflammatory myocardial injury—making it a non-specific signal in this context [4,13]. NT-proBNP adds prognostic information precisely because it captures the haemodynamic stress component—sustained RV wall stretch and pressure overload—that troponin alone cannot distinguish from other causes of cardiomyocyte injury.

A critical knowledge gap remains unaddressed in the current literature: no study has performed serial intraoperative NT-proBNP sampling during OLV with simultaneous pulmonary artery catheter monitoring to directly correlate real-time natriuretic peptide kinetics with haemodynamic parameters. Such a study would provide the first direct mechanistic evidence linking HPV-driven PVR changes to acute BNP gene transcription in humans and represents the most compelling target for future translational investigation in this field.

## 5. Preoperative NT-proBNP as a Predictor of Cardiopulmonary Complications

### 5.1. Evidence Base: Landmark Studies in Thoracic Surgery Populations

The evidence supporting preoperative NT-proBNP as a predictor of cardiopulmonary complications after lung resection derives from a coherent body of prospective studies, with the most influential conducted specifically in lung cancer surgery populations.

The landmark study in this field was published by Cardinale et al. in Circulation (2007), reporting a prospective cohort of 400 patients undergoing lung cancer surgery [45]. Perioperative NT-proBNP elevation was a powerful independent predictor of postoperative atrial fibrillation (POAF), with an incidence of 64% in patients with elevated preoperative NT-proBNP compared with 5% in those without elevation—an adjusted relative risk of 27.9 for preoperative elevation. This study remains the most cited in the field and established NT-proBNP as a clinically actionable preoperative biomarker in thoracic oncological surgery.

Complementary evidence from Nojiri et al., reported in two prospective studies in elderly lung cancer patients, established a BNP cut-off of 30 pg/mL (sensitivity 79%, specificity 83%, AUC 0.85) for predicting composite cardiopulmonary complications [14,46]. These investigators further proposed a three-tier risk stratification framework: patients with BNP below 30 pg/mL, between 30 and 100 pg/mL, and above 100 pg/mL experienced postoperative complication rates of 11%, 47%, and 85%, respectively—a strikingly linear dose–response that mirrors the pathophysiological gradient described in Section 4. It should be noted that both Nojiri studies measured BNP rather than NT-proBNP; given the approximately 5- to 10-fold difference in circulating concentrations between the two peptides, direct threshold translation is not appropriate without assay-specific recalibration.

Ellenberger et al. demonstrated in a mixed thoracic surgery cohort that elevated BNP was the strongest preoperative predictor of postoperative cardiovascular complications, with an odds ratio of 6.0, and further showed that BNP elevation was independently associated with reduced long-term survival after lung cancer surgery—extending the prognostic relevance of natriuretic peptide measurement beyond the immediate perioperative period [47]. This survival association was subsequently reinforced by Otsuka et al., who reported in 2026 that a preoperative BNP threshold of 18.5 pg/mL or above was associated with worse overall survival after curative lung cancer resection (HR 1.50), with the excess mortality driven predominantly by non-cancer-related deaths, suggesting that the biomarker captures cardiovascular rather than oncological risk [1].

From a predictive modelling perspective, Amar et al. developed and internally validated a BNP-based prediction model for POAF after thoracic surgery, achieving a C-statistic of 0.736 [36]. The model incorporated age, body mass index, BNP level, history of atrial fibrillation, and resection extent—confirming that natriuretic peptides contribute independent discriminative value even when clinical risk factors are accounted for.

### 5.2. Comparison with Standard Preoperative Risk Scores

The integration of NT-proBNP into formal preoperative risk assessment frameworks remains an area of active investigation and incomplete consensus. The 2024 AHA/ACC Perioperative Cardiovascular Evaluation Guidelines note that in the VISION substudy, preoperative NT-proBNP above 100 pg/mL was independently associated with all-cause mortality after non-cardiac surgery; however, the guidelines explicitly acknowledge that optimal threshold values for clinical decision-making are not yet clearly established [48]. Importantly, in a cohort of 3597 patients, the addition of NT-proBNP to the Revised Cardiac Risk Index (RCRI) did not significantly improve risk prediction beyond that achieved by RCRI combined with self-reported functional status—a finding that tempers enthusiasm for biomarker-based approaches in unselected surgical populations [48].

For thoracic surgery specifically, the Thoracic Revised Cardiac Risk Index (ThRCRI) has been validated as a population-specific risk tool incorporating variables most relevant to lung resection—including pneumonectomy, FEV_1_ below 40% predicted, ischaemic heart disease, and cerebrovascular disease—and demonstrates superior calibration to the generic RCRI in this setting [2,9]. However, the ThRCRI does not incorporate biomarker data, and no study has yet conducted a prospective head-to-head comparison of NT-proBNP against ThRCRI, DASI, or ACS-NSQIP calculators in a dedicated thoracic surgery population. Whether natriuretic peptide measurement adds meaningful incremental value to ThRCRI-based risk stratification—particularly in patients identified as intermediate risk—remains an unanswered question of direct clinical relevance.

### 5.3. The Cut-Off Problem: Absence of NT-proBNP-Specific Thresholds

A fundamental limitation of the current evidence base is the absence of a validated, assay-specific NT-proBNP cut-off for the thoracic surgery setting. The most widely cited thresholds—Nojiri’s 30 pg/mL and the three-tier framework—derive from BNP assays and are presented in Table 1 [46]. The 100 pg/mL threshold cited in the 2024 AHA/ACC guidelines originates from large general surgical cohorts rather than thoracic surgery populations [48]. Given that NT-proBNP concentrations are substantially higher than BNP concentrations in the same patient due to differences in clearance and half-life, and that NT-proBNP values are additionally confounded by age, sex, renal function, and obesity—as described in Section 3—applying BNP-derived cut-offs to NT-proBNP measurements without assay-specific recalibration risks both under- and over-classification of risk in clinical practice.

The development of an NT-proBNP cut-off validated specifically for pulmonary resection populations, ideally incorporating age- and sex-specific adjustments and stratified by resection extent, represents one of the most clinically pressing evidence gaps in this field. Until such data are available, NT-proBNP measurement in the preoperative thoracic surgery setting should be interpreted in the context of the individual patient’s clinical risk profile, renal function, and planned resection extent rather than against a single universal threshold.

## 6. Intraoperative NT-proBNP Dynamics

### 6.1. State of the Evidence

The intraoperative phase represents the most conspicuous gap in the perioperative NT-proBNP literature. No published study has performed serial NT-proBNP sampling during one-lung ventilation with simultaneous haemodynamic monitoring—a design that would allow direct correlation of real-time natriuretic peptide kinetics with pulmonary artery pressures, cardiac output, and RV function during the critical period of OLV-induced afterload mismatch. This absence is not incidental: it reflects the practical challenges of intraoperative biomarker sampling in the thoracic surgery setting, where arterial line access, anaesthetic management, and surgical exposure compete for clinical attention, and where the characteristic 12–24 h delay between haemodynamic stress and peak NT-proBNP elevation—a consequence of the burst-synthesis mechanism described in Section 4—limits the discriminatory value of a single intraoperative measurement.

### 6.2. Resection Extent as a Surrogate for Intraoperative Haemodynamic Load

The most relevant prospective dataset comes from Alonso et al., whose protocol and completed results measured high-sensitivity troponin and natriuretic peptides at preoperative and 24/48-h postoperative time points, deliberately omitting intraoperative sampling [4,13]. Although the study cannot address intraoperative kinetics, its completed results are informative for understanding the surgical determinants of perioperative myocardial stress: longer surgical duration—3.5 versus 2.7 h—and resection extent involving lobectomy or bilobectomy were independently associated with higher rates of perioperative myocardial injury, suggesting that cumulative intraoperative stress, rather than any discrete intraoperative event, drives postoperative biomarker elevation [27]. This finding implies that the intraoperative haemodynamic burden of major resection is already encoded in the early postoperative natriuretic peptide signal, even if it cannot be directly captured during the procedure itself.

The graded relationship between resection extent and postoperative atrial fibrillation risk, quantified by Amar et al., further supports this interpretation: pneumonectomy carried an OR of 6.70, oesophagectomy 4.93, and lobectomy 1.88 relative to segmentectomy as the reference procedure [36]. The dose–response pattern across resection types is consistent with a proportional increase in intraoperative pulmonary vascular bed reduction and RV afterload, the primary mechanistic drivers of NT-proBNP synthesis outlined in Section 4.

### 6.3. Knowledge Gaps and Priorities for Future Research

The intraoperative period therefore constitutes the most important unexplored domain in the perioperative NT-proBNP literature, with three specific knowledge gaps warranting targeted investigation.

First, no study has correlated intraoperative NT-proBNP or BNP concentrations with direct pulmonary artery pressure measurements during OLV or at the time of pulmonary artery clamping. Such a study—feasible in centres performing pulmonary artery catheterisation as part of routine thoracic anaesthesia practice—would provide the first mechanistic evidence directly linking HPV-driven PVR increases to natriuretic peptide transcription kinetics in humans, and would establish whether intraoperative sampling adds predictive value beyond preoperative baseline measurement.

Second, no comparative data exist on NT-proBNP dynamics between video-assisted thoracoscopic surgery (VATS) and open thoracotomy. Given the established differences in inflammatory response, surgical trauma, fluid shifts, and postoperative pain between the two approaches, it is plausible that VATS is associated with an attenuated natriuretic peptide response—but this hypothesis has not been directly tested with serial biomarker sampling.

Third, the impact of intraoperative ventilatory strategy on NT-proBNP release has not been prospectively studied. As reviewed in Section 4, pressure-controlled ventilation produces significantly better RV systolic function than volume-controlled ventilation during OLV [35], and PEEP titration meaningfully modulates RV afterload [31]. Whether these differences in intraoperative RV loading translate into measurable differences in postoperative NT-proBNP trajectories remains unknown and represents a compelling target for anaesthesia-focused research in this field.

## 7. Postoperative NT-proBNP as a Predictor of Outcomes

### 7.1. Postoperative Atrial Fibrillation

Postoperative atrial fibrillation (POAF) is the most extensively studied outcome in the perioperative NT-proBNP literature for thoracic surgery, and the evidence base is both consistent and clinically compelling. Cardinale et al. established that both preoperative and postoperative NT-proBNP elevations independently predicted POAF, with relative risks of 27.9 and 20.1 respectively—effect sizes that are rarely encountered in perioperative biomarker research and that reflect the tight mechanistic coupling between RV pressure overload and atrial stretch described in Section 4 [45]. The persistence of predictive value at both time points is clinically significant: it implies that NT-proBNP measurement adds prognostic information not only in the preoperative risk stratification window but also in the early postoperative period when clinical reassessment is still possible and prophylactic intervention remains feasible.

At the population level, a meta-analysis by Cai et al. encompassing 1844 patients across ten studies reported a pooled sensitivity of 75% and specificity of 80% for natriuretic peptides in predicting postoperative AF after thoracic surgery [12]. Two findings from this meta-analysis are particularly noteworthy for guiding clinical practice: NT-proBNP demonstrated superior discriminative performance compared to BNP, consistent with its longer half-life and greater stability as a perioperative biomarker; and postoperative assessment outperformed preoperative measurement, suggesting that the haemodynamic stress of surgery itself—RV afterload increase, pulmonary vascular bed reduction, and inflammatory activation—generates additional predictive signal beyond what is captured by the preoperative baseline. Toufektzian et al. further refined the postoperative threshold in a dedicated cohort, demonstrating that an NT-proBNP concentration of 113 pg/mL or above on the first postoperative day conferred an eightfold increased risk of POAF, providing a clinically applicable cut-off for early postoperative risk stratification [49].

### 7.2. Biomarker-Guided Atrial Fibrillation Prophylaxis: The PRESAGE Trial

The most clinically consequential study in this field is the PRESAGE trial, reported by Cardinale et al. which represents the only randomised controlled trial to demonstrate that NT-proBNP-guided prophylaxis can meaningfully reduce postoperative atrial fibrillation after lung cancer surgery [3]. Among 1116 patients undergoing lung cancer resection, 320 (29%) had elevated preoperative NT-proBNP and were randomised to metoprolol, losartan, or control. The reduction in POAF incidence was striking: 6% in the metoprolol arm, 12% in the losartan arm, and 40% in the control group (*p* < 0.001).

These findings, while striking, derive from a single-institution trial conducted predominantly in patients undergoing open thoracotomy, reflecting recruitment prior to the widespread adoption of video-assisted and robot-assisted thoracoscopic approaches; their generalisability to contemporary, minimally invasive, multi-institutional practice has not been established and should not be assumed. Beyond its primary finding, PRESAGE established a proof-of-concept of fundamental importance: NT-proBNP can function not merely as a passive prognostic marker but as an actionable triage tool—identifying the subset of patients who will derive the greatest benefit from targeted pharmacological prophylaxis and sparing low-risk patients from unnecessary treatment. The 29% prevalence of elevated preoperative NT-proBNP in this unselected lung cancer cohort is itself clinically informative, suggesting that nearly one in three patients undergoing pulmonary resection carries a modifiable haemodynamic risk that standard clinical assessment alone would not identify [3].

### 7.3. Composite Cardiopulmonary Complications

Beyond atrial fibrillation, postoperative natriuretic peptide elevation is predictive of a broader spectrum of cardiopulmonary complications. Cagini et al. demonstrated that a postoperative day 1 BNP concentration of 118.5 pg/mL or above was associated with a threefold increase in the risk of composite postoperative complications (OR 2.94), with the first postoperative day emerging as the optimal sampling time point—consistent with the burst-synthesis kinetics described in Section 4 and the 12–24 h delay between haemodynamic stress and peak peptide elevation [27]. The convergence of this BNP-based threshold with the NT-proBNP cut-off identified by Toufektzian et al. (113 pg/mL) at the same postoperative time point strengthens confidence in postoperative day 1 as the clinically optimal assessment window, notwithstanding the assay-specific nature of the individual thresholds [49]. Nojiri et al. confirmed a dose–response relationship between preoperative BNP tier and both complication severity and mortality, with the three-tier framework described in Section 5 demonstrating escalating event rates across the low, intermediate, and high-risk groups [14,46].

### 7.4. Major Adverse Cardiovascular Events and Perioperative Mortality

The combined assessment of natriuretic peptides and high-sensitivity troponin provides superior risk stratification to either biomarker alone in the postoperative period. Alonso et al. demonstrated that dual elevation in high-sensitivity cardiac troponin I and NT-proBNP postoperatively identified the highest-risk group with a MACE rate of 18.4%, compared with 3.3% in patients without perioperative myocardial injury—a more than fivefold difference. Postoperative mortality followed a similar gradient: 3.6% in the dual-elevation group versus 0.7% in those without biomarker elevation [4]. The complementary nature of these two biomarkers reflects the distinct but overlapping pathophysiological processes they capture: high-sensitivity troponin detects cardiomyocyte injury—whether ischaemic, inflammatory, or demand-mediated—while NT-proBNP reflects the haemodynamic stress component of sustained RV wall stretch and pressure overload. Together, they characterise both the injury and its haemodynamic context, and their combination represents the most informative postoperative biomarker strategy currently supported by evidence in the thoracic surgery literature.

### 7.5. Long-Term Survival

The prognostic reach of perioperative natriuretic peptide measurement extends beyond the immediate postoperative period. Ellenberger et al. demonstrated that elevated preoperative BNP predicted poor survival for up to four years after lung cancer surgery, with the association holding after adjustment for oncological variables—suggesting that natriuretic peptide elevation captures a component of cardiovascular risk that is independent of tumour biology [47]. Otsuka et al. confirmed this survival association, reporting a hazard ratio of 1.50 for overall mortality in patients with preoperative BNP at or above 18.5 pg/mL after curative lung resection, with the excess mortality driven predominantly by non-cancer-related deaths [1]. This finding has direct implications for the framing of natriuretic peptide measurement in lung cancer surgery: it positions the preoperative biomarker assessment not merely as a predictor of perioperative complications but as a prognostic tool that identifies patients whose cardiovascular reserve may be insufficient to sustain long-term survival independent of their oncological trajectory, and in whom postoperative cardiovascular follow-up may be warranted beyond the standard surgical care pathway.

### 7.6. Evidence Gaps in Postoperative Monitoring

Despite the strength of the evidence linking postoperative NT-proBNP to atrial fibrillation, MACE, and long-term survival, important gaps remain in the outcome-specific literature. Data directly linking postoperative NT-proBNP trajectories to right ventricular dysfunction following pneumonectomy—as distinct from the cardiac MRI-based evidence reviewed in Section 4—are limited, and no study has prospectively evaluated whether serial postoperative NT-proBNP monitoring can identify patients developing clinically significant RV-PA uncoupling before symptoms emerge. Similarly, the relationship between postoperative NT-proBNP elevation and pulmonary complications—including prolonged air leak, postpneumonectomy pulmonary oedema, and acute lung injury—as distinct from cardiovascular complications, has not been systematically examined. Finally, optimal monitoring frequency and duration in the postoperative period remain undefined: current evidence supports a single postoperative day 1 measurement, but whether additional sampling at day 3 or 7 provides incremental value for identifying patients with sustained RV dysfunction or at risk of late POAF has not been prospectively evaluated. These gaps represent priority areas for prospective study design, ideally incorporating serial echocardiography and right heart catheterisation alongside natriuretic peptide sampling to establish the mechanistic basis of postoperative NT-proBNP trajectories.

## 8. NT-proBNP Compared to Other Perioperative Biomarkers

### 8.1. NT-proBNP Versus BNP

Although BNP and NT-proBNP are derived from the same precursor molecule and released in equimolar quantities, their perioperative performance characteristics differ in ways that favour NT-proBNP for clinical use in thoracic surgery. As established in Section 3, NT-proBNP’s substantially longer half-life—approximately 120 min versus 20 min for BNP—confers greater temporal stability, making it less susceptible to acute fluctuations caused by haemodynamic events occurring immediately before blood sampling, a particular advantage in the dynamic perioperative environment of thoracic surgery. NT-proBNP is also unaffected by neprilysin inhibition, ensuring assay reliability in patients receiving sacubitril/valsartan, a drug class increasingly prevalent in the cardiac comorbidity profile of lung cancer surgery candidates [18].

At the level of clinical evidence, the meta-analysis by Cai et al.—encompassing 1844 patients across ten studies—directly compared the predictive performance of BNP and NT-proBNP for postoperative atrial fibrillation and found NT-proBNP to demonstrate superior discriminative value [12]. Postoperative measurement outperformed preoperative assessment for both peptides, but the advantage of NT-proBNP over BNP was consistent across time points. These findings support NT-proBNP as the preferred natriuretic peptide assay for perioperative risk stratification in thoracic surgery when local laboratory infrastructure permits, though it should be acknowledged that the majority of the foundational dose–response and threshold data in this field—including Nojiri’s three-tier framework and the PRESAGE trial entry criterion—were generated using BNP assays, creating an evidence base that is not fully interchangeable with NT-proBNP-specific thresholds [12,18].

### 8.2. NT-proBNP and High-Sensitivity Cardiac Troponin: A Complementary Relationship

The most clinically important biomarker comparison in the contemporary thoracic surgery literature concerns the relationship between NT-proBNP and high-sensitivity cardiac troponin I (hs-cTnI), which capture mechanistically distinct but pathophysiologically related aspects of perioperative cardiac stress. Troponin elevation after lung resection reflects cardiomyocyte injury—whether caused by ischaemia, demand mismatch from perioperative tachycardia, direct inflammatory myocardial injury, or RV strain—while NT-proBNP reflects the haemodynamic stress component of sustained ventricular wall stretch and pressure overload. Because troponin elevation in this context may arise from non-ischaemic mechanisms, it lacks the mechanistic specificity that the term perioperative myocardial injury implies when applied to general surgical populations, and NT-proBNP provides the haemodynamic context necessary to interpret troponin elevation correctly [4,13].

Alonso et al. quantified this complementary relationship in a prospective cohort of 475 thoracic surgery patients [4]. Dual postoperative elevation in hs-cTnI and NT-proBNP occurred in 10.3% of patients and was associated with the highest MACE rate at 15.2%—representing a two- to threefold increase over single biomarker elevation alone. Isolated troponin elevation without NT-proBNP elevation carried an intermediate risk profile, while isolated NT-proBNP elevation without troponin similarly identified a distinct risk stratum. The hierarchical risk gradient across these four groups—dual negative, isolated troponin, isolated NT-proBNP, and dual positive—demonstrates that the two biomarkers provide non-redundant prognostic information, and that their combination characterises the perioperative cardiac risk landscape with a resolution that neither marker achieves independently.

### 8.3. NT-proBNP Versus Inflammatory and Emerging Biomarkers

Beyond natriuretic peptides and troponins, a widening array of perioperative biomarkers has been evaluated in surgical populations, including markers of inflammation (C-reactive protein, interleukin-6), vascular dysfunction (galectin-3, soluble ST2), and matrix remodelling. The 2024 ACC/AHA Appropriate Use Criteria document acknowledges that several of these emerging biomarkers have demonstrated incremental prognostic information over natriuretic peptides alone in general cardiac and surgical populations [50]. However, thoracic surgery-specific comparative data are conspicuously absent from the retrieved literature: no published study has conducted a head-to-head evaluation of NT-proBNP against CRP, galectin-3, sST2, or other candidate biomarkers in a dedicated lung cancer resection cohort. This gap is particularly relevant given the pronounced inflammatory component of thoracic surgery—ischaemia–reperfusion injury, one-lung ventilation-induced cytokine release, and mediastinal manipulation all generate systemic inflammatory signals that may confound the interpretation of inflammation-based biomarkers in ways that do not apply to elective cardiac or non-thoracic surgery populations.

Until thoracic surgery-specific comparative data are available, NT-proBNP combined with hs-cTnI represents the most evidence-based biomarker strategy for perioperative cardiac risk assessment in lung resection, with the dual biomarker approach offering superior risk stratification over either marker alone. The incremental value of adding inflammatory or fibrosis biomarkers to this core panel remains an open and clinically important research question.

## 9. Surgical Approach as a Modulator of Perioperative Cardiac Stress: The Emerging Evidence

### 9.1. Three Pathways of Influence

The surgical approach to pulmonary resection—open thoracotomy, video-assisted thoracoscopic surgery (VATS), or robot-assisted thoracoscopic surgery (RATS)—modulates perioperative NT-proBNP dynamics through three interconnected pathways: differential systemic inflammatory burden, differential haemodynamic stress during one-lung ventilation, and differential duration of OLV itself. The relationship between surgical invasiveness and cardiac biomarker elevation is, however, considerably more complex than a simple hierarchy of trauma would predict—a complexity that has important implications for the interpretation of postoperative NT-proBNP thresholds across contemporary thoracic surgery practice.

### 9.2. VATS Versus Open Thoracotomy: Consistent Reduction in Postoperative Atrial Fibrillation

The evidence that minimally invasive lung resection reduces the incidence of postoperative atrial fibrillation and composite cardiopulmonary complications relative to open thoracotomy is robust and consistent across multiple study designs and databases. Analysis of the ESTS Database by Falcoz et al. in 2721 propensity-matched patients demonstrated that VATS lobectomy was associated with significantly lower rates of cardiopulmonary complications (15.9% vs. 19.6%, *p* = 0.0094) and lower mortality (1.0% vs. 1.9%, *p* = 0.0201) [51]. Villamizar et al. confirmed a significant reduction in POAF with VATS in 284 propensity-matched pairs (13% vs. 21%, *p* = 0.01), alongside lower rates of pneumonia and renal failure [52]. The meta-analysis by Ye and Wang, encompassing 15 studies, found consistent reductions in atrial arrhythmias, prolonged pneumonia, and renal failure with VATS compared to thoracotomy [53].

More recent data extend the temporal horizon of this benefit. Woolard et al., in a 2026 cohort of 899 patients, reported POAF incidence of 13.0% after open thoracotomy versus 6.5% after VATS (*p* = 0.001), and demonstrated that POAF was independently associated with prolonged ICU stay (4.68 vs. 3.10 days, *p* = 0.009), increased reintubation rates (*p* < 0.001), and higher rates of return to the operating room (*p* = 0.003)—quantifying the downstream clinical consequences of approach-related atrial fibrillation risk [54]. Halloran et al., analysing 22,998 patients from the TriNetX database with propensity matching of 7942 pairs, demonstrated not only higher early POAF with open thoracotomy (5.5% vs. 3.4%, RR 1.607, *p* < 0.001) but also higher rates of chronic POAF at 12 to 24 months (6.3% vs. 5.2%, RR 1.211, *p* = 0.003)—a finding that implies surgical approach has lasting effects on atrial substrate remodelling beyond the acute perioperative period [55].

### 9.3. The Inflammatory Pathway: How Surgical Approach Modulates Cytokine-Mediated Cardiac Stress

The mechanistic basis for reduced cardiac complications with minimally invasive approaches is at least partially attributable to an attenuated systemic inflammatory response, which in turn reduces cytokine-mediated stimulation of BNP gene transcription. As established in Section 4, IL-6 and TNF-α directly stimulate natriuretic peptide synthesis in cardiomyocytes independent of haemodynamic effects—meaning that the inflammatory burden of surgical trauma constitutes an independent, non-haemodynamic stimulus for postoperative NT-proBNP elevation.

Yim et al. demonstrated in 36 patients that VATS lobectomy produced significantly lower postoperative IL-6 and IL-8 concentrations compared to open thoracotomy, alongside reduced IL-10—indicating an overall attenuated inflammatory cascade rather than a selective suppression of any single cytokine [56]. Menna et al. extended this finding to the wound-site microenvironment in 30 patients, demonstrating fewer polymorphonuclear cells (*p* = 0.001), a decreased granulocyte fraction (*p* = 0.01), and lower systemic IL-1β (*p* = 0.038) after VATS compared to thoracotomy, confirming that the inflammatory difference propagates from the local surgical site to the systemic circulation [57]. Zhang et al. further showed in 122 patients that VATS produced significantly lower postoperative WBC counts, high-sensitivity CRP, VEGF, and IL-6 at all postoperative time points, with better-preserved CD3+, CD4+, and CD8+ T-lymphocyte counts, indicating less immunosuppression alongside reduced inflammation [58].

Despite this mechanistic plausibility, no published study has directly measured NT-proBNP in a prospective VATS versus open thoracotomy comparison with serial perioperative sampling. The inference that reduced cytokine burden after VATS translates into attenuated NT-proBNP elevation—while biologically compelling—remains unvalidated at the biomarker level and constitutes one of the most important evidence gaps in this field.

### 9.4. RATS Versus VATS: A Paradox in Cardiac Biomarker Data

The comparison between robot-assisted and standard video-assisted thoracoscopy reveals an unexpected finding that directly challenges the intuitive assumption that greater minimally invasive refinement produces proportionally less cardiac stress. Alonso et al., in the most important study to date directly measuring cardiac biomarkers across surgical approaches in 475 thoracic surgery patients, demonstrated that RATS was associated with a significantly increased risk of perioperative myocardial injury compared to VATS (OR 2.29, *p* = 0.019) [4]. Among patients with dual elevation in hs-cTnI and NT-proBNP—the highest-risk group, with a MACE rate of 18.4%—the majority had undergone minimally invasive procedures (VATS 61.2%, RATS 24.5%), with a median operative duration of 3 h and 42 min.

This paradox—RATS producing more cardiac biomarker elevation than VATS despite comparable or superior tissue preservation—can be explained by four converging factors. First, RATS consistently requires longer operative times than VATS: Catelli et al. reported a median difference of 20 min (180 vs. 160 min, *p* = 0.036) and Ichinose et al. a difference of 26 min (199 vs. 173 min, *p* < 0.001), translating directly into longer OLV duration and therefore more sustained RV afterload stress [59,60]. Second, the inflammatory profiles of the two approaches are counterintuitively divergent: Jaradeh et al. demonstrated in 45 patients that VATS produced significantly higher IL-6 (*p* < 0.001) and MCP-1 (*p* = 0.005) concentrations at two hours postoperatively compared to RATS, with higher MCP-1 persisting at 24 h (*p* < 0.001) [61]—a finding confirmed by Hong et al. in 276 propensity-matched patients, who reported lower CRP, IL-6, and better-preserved T-lymphocyte subsets after RATS compared to VATS [62]. The fact that RATS induces less systemic inflammation than VATS yet produces more myocardial injury strongly suggests that the haemodynamic mechanism—specifically OLV duration-dependent RV afterload mismatch—dominates over the inflammatory pathway in determining cardiac biomarker elevation. Third, RATS frequently involves CO_2_ insufflation into the hemithorax to optimise visualisation, increasing intrathoracic pressure and further compromising venous return and RV preload—an additional haemodynamic stressor absent from standard VATS. Fourth, patient selection and institutional learning curve effects may contribute, as centres performing RATS tend to accept more complex cases with longer anticipated operative times.

### 9.5. OLV Duration as the Dominant Determinant: A Unifying Framework

The RATS paradox, interpreted alongside the surgical duration data from Alonso et al.—where longer surgery (3.5 vs. 2.7 h, *p* < 0.001) was independently associated with perioperative myocardial injury regardless of approach [4]—suggests that OLV duration may be the single dominant determinant of postoperative NT-proBNP elevation, superseding the inflammatory differences between surgical approaches. This framework is biologically coherent: as reviewed in Section 4, compensatory mechanisms including HPV optimisation and Frank-Starling reserve become progressively exhausted during sustained OLV, and Rana et al. have conceptualised the resulting cumulative myocardial damage as “RV injury”—a duration-dependent phenomenon that predisposes to acute dysfunction [6]. Ventilatory strategy during OLV further modulates this exposure: pressure-controlled ventilation preserves RV systolic function more effectively than volume-controlled ventilation during OLV, as demonstrated by Al Shehri et al. [33], suggesting that the haemodynamic cardiac stress of any given surgical approach is substantially modified by anaesthetic management independent of the approach itself.

### 9.6. Implications for NT-proBNP Threshold Interpretation

The evidence reviewed in this section has three direct implications for the clinical interpretation of perioperative NT-proBNP values in contemporary thoracic surgery. First, postoperative NT-proBNP thresholds may require approach-specific calibration: if RATS produces higher NT-proBNP elevations than VATS primarily due to longer OLV duration rather than intrinsically greater cardiac risk, a single threshold applied across all approaches risks systematically misclassifying RATS patients as higher risk than their true cardiovascular status warrants. The OR of 2.29 for RATS-associated perioperative myocardial injury reported by Alonso et al. should therefore be interpreted with appropriate caution as a potential surrogate for operative duration rather than a measure of approach-specific surgical trauma [4]. Second, the PRESAGE trial threshold of 182.3 ng/L was derived predominantly from open thoracotomy patients, given that the trial was conducted between 2007 and 2013 when VATS adoption was substantially lower than in contemporary practice [3,15]. Its applicability to contemporary VATS and RATS populations has not been validated and should not be assumed. Third, and importantly, preoperative NT-proBNP retains its predictive value regardless of the planned surgical approach, since it reflects baseline cardiac reserve rather than the stress response to surgery—a property confirmed by the Bu et al. nomogram, which incorporated preoperative NT-proBNP alongside surgical variables including lobectomy type in a VATS-specific population [63].

A final methodological consideration concerns the potential superiority of delta NT-proBNP—the change from preoperative baseline to postoperative measurement—over absolute postoperative values. Cagini et al. demonstrated that patients who developed complications had a significantly greater BNP increase from baseline compared to those without complications (*p* < 0.0001), independent of baseline values [27], suggesting that the perioperative increment captures the surgical stress response more precisely than any single postoperative threshold. Whether delta NT-proBNP provides superior risk stratification across surgical approaches—and whether approach-specific reference change values can be established—represents a compelling methodological priority for future research.

### 9.7. Evidence Gaps and Research Priorities

Five specific knowledge gaps warrant targeted investigation. No study has directly compared NT-proBNP kinetics across open, VATS, and RATS approaches in a prospective controlled design with serial intraoperative and postoperative sampling—this represents the single most important unanswered question in this section of the literature. The dose–response relationship between cumulative OLV duration and NT-proBNP elevation has never been formally quantified, and a study correlating OLV time with serial natriuretic peptide measurements would directly test the OLV duration hypothesis. Sublobar resections—segmentectomy and wedge resection—are increasingly performed via minimally invasive approaches and would be expected to produce less NT-proBNP elevation due to greater pulmonary vascular bed preservation, but no comparative biomarker data exist for sublobar versus lobar resections within the same approach. The paradox of RATS-associated perioperative myocardial injury versus RATS-associated lower atrial fibrillation risk requires resolution through larger multicentre studies controlling for OLV duration, comorbidity burden, and institutional learning curves. Finally, uniportal VATS—which produces even less immunochemokine disturbance than multiportal VATS—has never been evaluated for its impact on cardiac biomarkers [64], representing a natural experiment for isolating the contribution of the inflammatory pathway to postoperative NT-proBNP elevation. Building directly on the RATS paradox described above, a further priority is the prospective derivation and validation of approach-specific NT-proBNP thresholds—separately calibrated for open thoracotomy, VATS, and RATS—stratified by cumulative OLV duration rather than surgical approach alone; applying a single threshold across all three approaches risks systematically misclassifying patients whose biomarker elevation reflects prolonged ventilatory stress rather than greater intrinsic cardiac risk.

## 10. The Cut-Off Heterogeneity Problem

### 10.1. The Absence of a Universal Threshold

A central and unresolved challenge for the clinical implementation of perioperative NT-proBNP measurement in thoracic surgery is the absence of a single validated, universally applicable threshold. The 2024 AHA/ACC Perioperative Guidelines explicitly acknowledge that optimal threshold values for BNP and NT-proBNP in perioperative risk prediction are not clearly established [48]—a statement that reflects not a failure of research effort but the genuine complexity of applying a continuous physiological variable to heterogeneous populations, assays, outcomes, and clinical contexts. The proposed cut-offs in the thoracic surgery literature vary by an order of magnitude, from Nojiri’s BNP threshold of 30 pg/mL to NT-proBNP concentrations exceeding 1500 pg/mL in the VISION cohort, and no single value has been prospectively validated across more than one centre in a thoracic surgery-specific population.

### 10.2. Consolidated Thresholds in the Thoracic Surgery Literature

The principal thresholds identified in studies specific to lung cancer and thoracic surgery are summarised in Table 2. Several observations emerge from this consolidation. The Salvatici-derived cut-off of 182.3 ng/L, subsequently adopted by the PRESAGE trial, represents the only NT-proBNP threshold that has been used to guide a therapeutic intervention with demonstrated clinical efficacy—giving it unique practical relevance despite its derivation from a single centre [3,15]. The Nojiri BNP threshold of 30 pg/mL, validated across two prospective studies in elderly lung cancer patients with AUC values of 0.85 to 0.90, provides the strongest diagnostic performance data in the preoperative setting but is assay-specific to BNP and cannot be directly translated to NT-proBNP without recalibration [15,46]. The postoperative day 1 BNP threshold of 118.5 pg/mL identified by Cagini et al. is consistent with the burst-synthesis kinetics reviewed in Section 4, and its temporal specificity—first postoperative day—is clinically actionable [27]. The NT-proBNP threshold of 113 pg/mL reported by Toufektzian et al. for POAF prediction, derived from a non-cardiac thoracic surgery population, confers an eightfold increase in risk and aligns conceptually with the lower end of the intermediate risk zone proposed below [49].

### 10.3. Why Thresholds Diverge: A Systematic Analysis

Five distinct sources of heterogeneity account for the wide variation in proposed cut-offs, and their explicit recognition is essential for the correct interpretation of the published literature.

The first and most fundamental source is the non-interchangeability of BNP and NT-proBNP assays. Although the two peptides are released in equimolar quantities, circulating NT-proBNP concentrations are substantially higher than BNP concentrations in the same patient due to differences in clearance half-life and volume of distribution. A conversion factor of approximately five- to sixfold is sometimes applied—yielding an NT-proBNP equivalent of 150 to 180 pg/mL for Nojiri’s BNP threshold of 30 pg/mL—and the convergence of this estimated range with the Salvatici/PRESAGE threshold of 182.3 ng/L is notable, suggesting biological coherence across the two assay systems [15,46]. However, this conversion ratio varies substantially with age, renal function, and immunoassay platform, and should not be applied mechanically in clinical practice. Approximately half of the thoracic surgery studies reviewed here used BNP assays (Nojiri, Cagini, Amar) and half used NT-proBNP (Cardinale, Salvatici, Alonso), creating a bifurcated evidence base that cannot be directly synthesised without assay-specific adjustment.

The second source of heterogeneity is outcome specificity. Cut-offs optimised for POAF prediction are consistently lower than those optimised for MACE or mortality, reflecting the biological reality that atrial fibrillation occurs at lower levels of haemodynamic stress than myocardial infarction or death. The Salvatici and Nojiri thresholds—derived for POAF—sit in the range of 30 to 182 pg/mL equivalent, while MACE-optimised thresholds from general surgical cohorts begin at 300 pg/mL and above [15,46]. Applying a POAF-derived cut-off to MACE prediction, or vice versa, will produce systematically miscalibrated risk estimates.

The third source is measurement timing. Postoperative thresholds are physiologically higher than preoperative thresholds because surgery itself—through OLV-induced RV afterload, ischaemia–reperfusion injury, volume shifts, and sympathetic activation—generates a natriuretic peptide rise that is superimposed on the preoperative baseline. Cagini’s postoperative day 1 BNP threshold of 118.5 pg/mL must therefore be interpreted relative to a surgically elevated baseline rather than against reference ranges derived from resting ambulatory populations [27]. his distinction also underlies the finding from Cai et al. that postoperative measurement outperforms preoperative assessment for POAF prediction: the postoperative value integrates both the preoperative cardiac reserve and the surgical haemodynamic stress response into a single measurement [12].

The fourth source is population composition. The VISION cohort—from which the 100 pg/mL and 1500 pg/mL thresholds derive—comprised predominantly orthopaedic (28%), abdominal (24%), and vascular (8%) surgery patients, with thoracic surgery representing a minority subgroup [65]. Thoracic surgery patients face haemodynamic stressors—OLV, pulmonary vascular bed reduction, mediastinal manipulation, and wave reflection—that are not represented in general surgical cohorts and that may shift the optimal threshold in directions that cannot be predicted from non-thoracic data alone. The application of general surgical thresholds to thoracic surgery populations therefore carries an unquantified but potentially clinically significant risk of miscalibration.

The fifth, and most underappreciated, source of heterogeneity is immunoassay platform variability. Different platforms—Roche Elecsys, Abbott Architect, Siemens Dimension—yield systematically different absolute NT-proBNP values, particularly at lower concentrations where the measurement uncertainty is proportionally greatest. The Rodseth meta-analysis and VISION study used the Roche Elecsys platform, while other thoracic surgery studies used varying platforms without explicit cross-calibration [66]. This assay-level heterogeneity operates silently beneath the clinical literature and means that numerical thresholds derived on one platform may not be reproducible on another without platform-specific validation.

### 10.4. Toward a Pragmatic Tiered Framework

Pending prospective multicentre validation of a thoracic surgery-specific NT-proBNP threshold, the evidence supports a pragmatic four-tier risk framework based on the convergence of available data across outcomes and populations.

Concentrations below 100 pg/mL represent a consistently low-risk zone: event rates below 5% for both MACE and POAF have been reported across all studies using thresholds in this range, and the negative predictive value in this zone is robust and supported by the VISION data [65,66]. Concentrations between 100 and 300 pg/mL constitute an intermediate or grey zone in which risk is modestly but meaningfully elevated. The Salvatici and PRESAGE threshold of 182.3 ng/L falls within this range and, as the only cut-off validated in an interventional trial with demonstrated efficacy, carries particular clinical relevance for POAF prophylaxis decision-making [3,15]. Concentrations above 300 pg/mL represent a high-risk zone consistently associated with substantially elevated rates of MACE, mortality, and POAF across general surgical and thoracic cohorts, and supported by the Rodseth meta-analysis, VISION data, and AHA/ACC guidelines [48,67]. Concentrations at or above 1500 pg/mL define a very high-risk zone in which 30-day mortality reaches approximately 4.0% and MACE incidence 37.5% in the VISION cohort [65], and in which the appropriateness of proceeding with elective pulmonary resection warrants multidisciplinary reassessment.

This tiered framework is explicitly intended as a clinical decision support tool rather than a validated diagnostic algorithm. Its thresholds are derived from NT-proBNP assays and are not interchangeable with BNP values without the conversion caveats described above. The framework also does not account for the approach-specific calibration issues identified in Section 9, nor for the confounding effects of age, renal function, obesity, and pre-existing atrial fibrillation described in Section 3—all of which should inform the interpretation of any individual measurement.

From a health economic perspective, Devereaux et al. have argued that natriuretic peptide measurement is preferable to stress testing in the preoperative setting because it provides superior predictive accuracy at lower cost, with results available within minutes using point-of-care platforms [66]. This cost-effectiveness argument strengthens the case for systematic preoperative NT-proBNP measurement in thoracic surgery candidates, even in the absence of a validated single threshold, provided that the result is interpreted within a structured clinical framework rather than against an isolated numerical cut-off.

### 10.5. The BNP-to-NT-proBNP Conversion Problem: A Call for Standardisation

The literature’s bifurcation between BNP and NT-proBNP assays is not merely a technical inconvenience—it represents a fundamental barrier to evidence synthesis that has prevented the field from converging on a unified clinical standard. A multicentre prospective study using standardised NT-proBNP assays, predefined thresholds stratified by resection extent and surgical approach, and prespecified outcome definitions would resolve the most important methodological limitation in the current evidence base. Until such data are available, clinicians should be explicit about which assay they are using when interpreting published thresholds and should not apply BNP-derived cut-offs to NT-proBNP measurements or vice versa without acknowledging the inherent approximation this entails.

## 11. The Dual Biomarker Strategy: An Emerging Paradigm

### 11.1. Beyond Single-Threshold Strategies

The limitations of single-biomarker, single-threshold approaches described in Section 10—assay heterogeneity, outcome specificity, timing dependence, and population variability—point toward a more nuanced perioperative risk stratification framework. The emerging evidence suggests that the simultaneous assessment of two mechanistically complementary biomarkers may provide risk discrimination that neither can achieve independently, and that the pattern of biomarker elevation—rather than any individual numerical threshold—carries the greatest prognostic signal.

The biological rationale for a dual biomarker approach rests on the distinct but overlapping pathophysiological processes captured by high-sensitivity cardiac troponin I and NT-proBNP in the thoracic surgery setting. As established in Section 2 and Section 6, hs-cTnI reflects cardiomyocyte injury—from ischaemia, demand mismatch, RV strain, or direct inflammatory myocardial damage—while NT-proBNP reflects sustained haemodynamic stress through ventricular wall stretch and pressure overload. These two processes are related but non-redundant: a patient may sustain significant RV pressure overload after pneumonectomy without cardiomyocyte injury detectable by troponin, while conversely a patient may develop demand-related troponin elevation during tachycardia without the sustained RV afterload excess that drives NT-proBNP synthesis. The combination of the two biomarkers therefore characterises both the injury and its haemodynamic context simultaneously, providing a two-dimensional view of perioperative cardiac stress that a single biomarker cannot replicate.

### 11.2. Evidence from the Alonso et al. [4] Multicentre Study

The most important dataset directly supporting a dual biomarker strategy in thoracic surgery comes from Alonso et al., whose 2026 multicentre study in 475 patients established a hierarchical four-group risk classification based on the postoperative pattern of hs-cTnI and NT-proBNP elevation [4]. Patients with dual elevation in both biomarkers—representing 10.3% of the cohort—experienced a MACE rate of 18.4% and a 30-day mortality of 3.6%, representing a five- to sixfold increase over patients without perioperative myocardial injury (MACE 3.3%, mortality 0.7%). Isolated troponin elevation and isolated NT-proBNP elevation each defined intermediate risk strata, with MACE rates falling between the dual-negative and dual-positive extremes. This hierarchical gradient—with each additional biomarker elevation incrementally increasing risk—demonstrates that the two markers provide additive rather than redundant prognostic information, and that their combination produces a risk classification with greater clinical resolution than either threshold alone [4].

The surgical approach distribution within the dual-elevation group is itself informative: 61.2% of dual-elevation patients had undergone VATS and 24.5% RATS, with a median operative duration of 3 h and 42 min—reinforcing the OLV duration hypothesis advanced in Section 9 and suggesting that the dual biomarker signal is generated predominantly by sustained haemodynamic stress rather than by the tissue trauma of open surgery per se [4].

### 11.3. A Proposed Clinical Decision Framework

**Figure 4:**
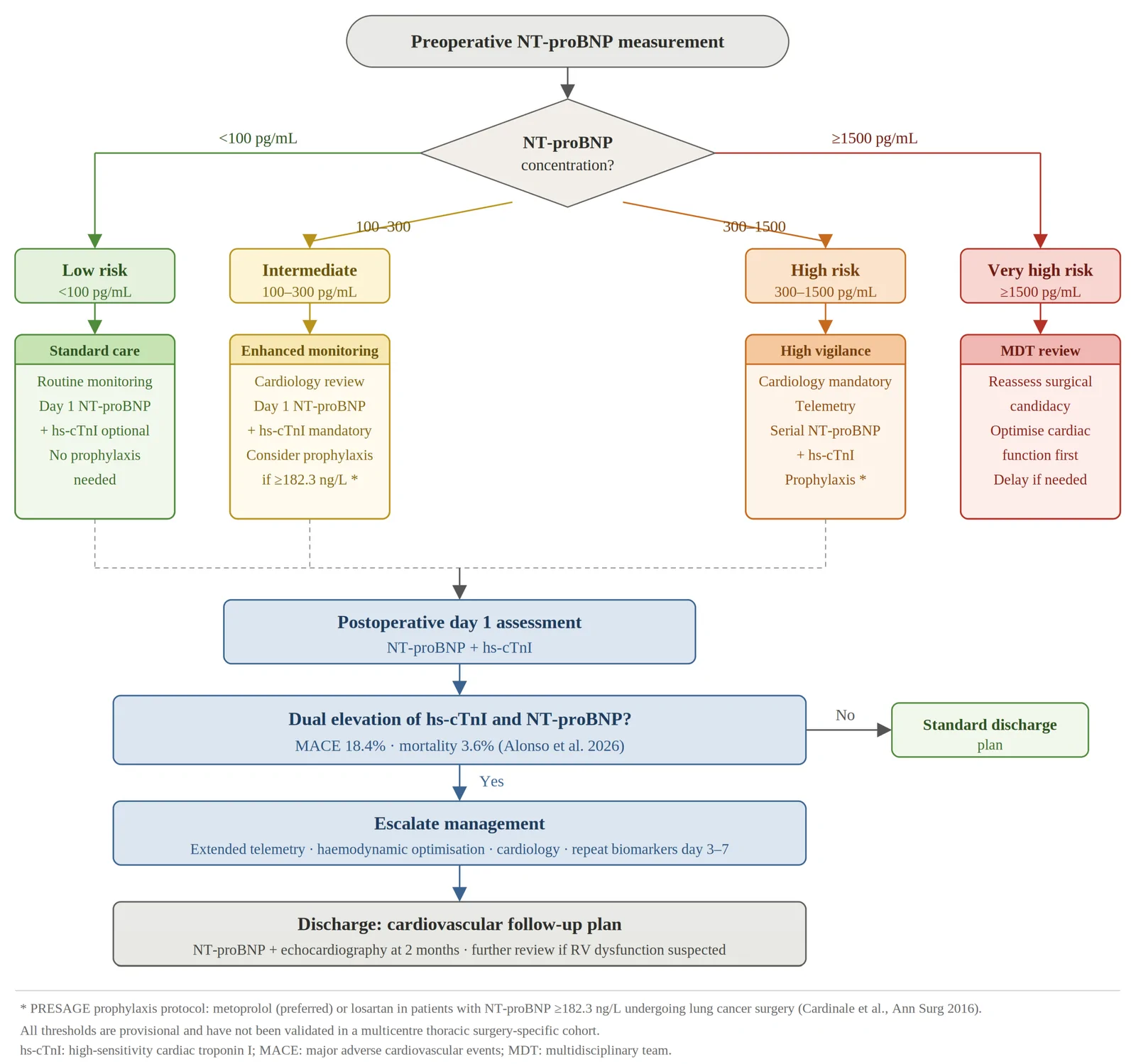
Proposed clinical algorithm for perioperative NT-proBNP-guided risk stratification and monitoring in lung cancer surgery. Preoperative NT-proBNP concentration is used to stratify patients into four risk tiers, each associated with specific perioperative management recommendations Colours denote escalating risk according to the conventional clinical gradient: green (low risk), yellow (intermediate risk), orange (high risk), and red (very high risk); blue boxes indicate the shared postoperative assessment and management pathway, and grey boxes indicate the entry and discharge steps. Tiers converge at a postoperative day 1 dual biomarker assessment; dual elevation of NT-proBNP and hs-cTnI identifies the highest-risk subgroup (Alonso et al., 2026 [4]). The prophylaxis recommendation follows the PRESAGE protocol (Cardinale et al., 2016 [3]). hs-cTnI, high-sensitivity cardiac troponin I; MACE, major adverse cardiovascular events; MDT, multidisciplinary team; NT-proBNP, N-terminal pro-B-type natriuretic peptide.

Integrating the dual biomarker evidence with the tiered NT-proBNP framework proposed in Section 10, a pragmatic postoperative monitoring algorithm can be outlined for discussion. Patients with postoperative NT-proBNP below 100 pg/mL and no troponin elevation constitute a low-risk group in whom standard postoperative care is appropriate. Patients with NT-proBNP in the intermediate zone of 100 to 300 pg/mL—particularly those with concurrent troponin elevation—warrant enhanced cardiac monitoring, rhythm surveillance for POAF, and consideration of the prophylactic strategies validated in the PRESAGE trial framework. Patients with NT-proBNP above 300 pg/mL, particularly when accompanied by hs-cTnI elevation, represent the highest-risk group in whom cardiology consultation, extended telemetry, and aggressive haemodynamic optimisation are warranted. The PRESAGE trial demonstrates that this highest-risk group—identifiable preoperatively by NT-proBNP alone—can benefit from targeted pharmacological prophylaxis, with metoprolol reducing POAF incidence from 40% to 6% in NT-proBNP-elevated patients [3]. The proposed perioperative management pathway integrating preoperative risk stratification with postoperative dual biomarker assessment is summarised in Figure 4.

This framework is explicitly provisional: it represents a synthesis of available evidence rather than a prospectively validated algorithm, and its thresholds carry all the caveats regarding assay specificity, timing, and population heterogeneity detailed in Section 10. Its primary purpose is to provide clinicians with a structured approach to biomarker interpretation while the field moves toward the multicentre prospective validation studies that the evidence base urgently requires. Multicentre prospective validation is not merely desirable but should be regarded as a prerequisite for clinical standardisation: until the tiered thresholds proposed here are tested against hard cardiovascular endpoints in adequately powered, contemporary, multi-institutional cohorts, this framework should inform clinical judgement rather than replace it and should not be adopted as a fixed diagnostic algorithm in routine practice.

### 11.4. The Graded, Continuous Nature of NT-proBNP Risk

The graded, continuous nature of this dose–response relationship—synthesised from key thoracic surgery datasets—is illustrated in Figure 3. An important conceptual refinement emerging from the broader perioperative biomarker literature—exemplified by Pölzl et al. in cardiac surgery populations—is that the relationship between preoperative NT-proBNP and postoperative adverse outcomes is graded and continuous rather than binary [68]. Risk does not switch abruptly at any single threshold; it increases progressively across the entire range of NT-proBNP concentrations, with each incremental elevation carrying additional prognostic weight. This continuous dose–response relationship—demonstrated in the thoracic surgery context by the linear escalation of complication rates across Nojiri’s three tiers (11%, 47%, 85%) and the graded MACE gradient across Alonso’s four biomarker groups—has two clinical implications. First, a patient with an NT-proBNP of 180 pg/mL should not be regarded as categorically different from one at 200 pg/mL simply because a threshold of 182.3 ng/L has been proposed; clinical judgement applied to the full clinical context remains essential. Second, the delta NT-proBNP—the perioperative increment from preoperative baseline—may ultimately prove more informative than any absolute postoperative value, as it isolates the surgical stress response from the patient’s chronic cardiac loading state and controls for the baseline confounders of age, sex, renal function, and obesity described in Section 3.

## 12. Discussions

The evidence reviewed across the preceding sections supports a coherent, if incomplete, picture of NT-proBNP as a perioperative biomarker in lung cancer surgery. Several overarching themes emerge from this synthesis that warrant explicit discussion.

The first and most clinically consequential is the absence of a validated, thoracic surgery-specific NT-proBNP threshold. This review has identified at least eight distinct cut-off values in the thoracic surgery literature, ranging from Nojiri’s BNP threshold of 30 pg/mL to NT-proBNP concentrations exceeding 1500 pg/mL in general surgical cohorts, with no single value prospectively validated across more than one centre in a dedicated pulmonary resection population [14,46,48]. The Salvatici threshold of 182.3 ng/L comes closest to this standard—it was derived from a prospective cohort of 400 lung cancer surgery patients and subsequently adopted as the entry criterion for the PRESAGE trial—but its single-centre origin limits its generalisability to populations with different case-mix, surgical approach distribution, and comorbidity burden [3,15]. The PRESAGE trial itself, conducted between 2007 and 2013 when open thoracotomy predominated, may not accurately represent the contemporary thoracic surgery landscape in which VATS and RATS account for the majority of resections at high-volume centres. The practical implication is that clinicians currently applying any published NT-proBNP threshold to guide perioperative decision-making are extrapolating beyond the validated evidence base and should do so explicitly and with appropriate clinical judgement rather than as a mechanical rule.

The second theme is the BNP-to-NT-proBNP conversion problem, which constitutes a structural barrier to evidence synthesis that has been systematically underacknowledged in the field. Approximately half of the thoracic surgery studies identified in this review used BNP assays—Nojiri, Cagini, and Amar [14,27,36]—and half used NT-proBNP—Cardinale, Salvatici, and Alonso [3,4,15]—without cross-assay calibration. The approximate fivefold to sixfold conversion ratio between the two peptides is biologically derived and contextually variable, and its application without assay-specific validation introduces a layer of numerical imprecision that compounds the other sources of threshold heterogeneity described in Section 10. Until the field converges on a standardised assay platform—ideally NT-proBNP, given its superior stability and freedom from neprilysin interference—meaningful meta-analytic synthesis of cut-off data will remain methodologically compromised.

The third theme is the emergence of the dual biomarker strategy as the most promising direction for perioperative risk stratification in thoracic surgery. The Alonso et al. data demonstrate that the combination of hs-cTnI and NT-proBNP provides a risk gradient—from 3.3% MACE in the dual-negative group to 18.4% in the dual-positive group [4]—that exceeds the discriminative capacity of either marker alone and that maps mechanistically onto the two distinct pathophysiological processes of myocardial injury and haemodynamic stress. This two-dimensional risk characterisation represents a conceptual advance over single-threshold strategies and aligns with the broader movement in perioperative medicine toward multi-marker panels for complex surgical risk assessment. Its limitation at present is that it derives from a single multicentre study, and independent validation in a separate thoracic surgery cohort is required before it can be recommended as a clinical standard [4].

The fourth theme, emerging from Section 9, is the previously unrecognised complexity introduced by surgical approach. The paradox of RATS-associated perioperative myocardial injury [4] despite lower systemic inflammation than VATS [61,62]—most plausibly explained by longer OLV duration [60] rather than greater surgical trauma—suggests that existing perioperative NT-proBNP thresholds, derived predominantly from open thoracotomy cohorts, may systematically misclassify patients undergoing contemporary minimally invasive resection. The OLV duration hypothesis advanced in this review—that cumulative RV afterload exposure during single-lung ventilation is the dominant determinant of postoperative NT-proBNP elevation, superseding the inflammatory differences between approaches—is biologically coherent and supported by converging lines of indirect evidence, but has not been directly tested in a prospective study with serial intraoperative biomarker sampling. It represents, in our assessment, the most important mechanistic hypothesis in this field awaiting formal evaluation.

The fifth theme concerns the temporal dimension of NT-proBNP monitoring. The multi-hit model of NT-proBNP elevation described in Section 4—spanning four phases from intraoperative OLV through chronic pulmonary vascular remodelling—implies that a single perioperative measurement captures only one moment in a dynamic and evolving process. The evidence from McCall et al. that RV dysfunction persists at two months [7], and from Tan et al. that RV-PA uncoupling predicts survival at three and five years [16], suggests that the prognostic horizon of perioperative NT-proBNP measurement extends far beyond what is conventionally regarded as the perioperative window. Whether systematic postoperative NT-proBNP surveillance—at hospital discharge, at two months, and at six months—can identify patients developing clinically significant chronic pulmonary hypertension or RV dysfunction before symptoms emerge, and whether early intervention in this group improves long-term outcomes, remains an entirely open question of considerable clinical importance.

Finally, the PRESAGE trial stands as both the field’s greatest achievement and its most pressing unfinished business. The demonstration that NT-proBNP-guided metoprolol prophylaxis reduces POAF from 40% to 6% in high-risk thoracic surgery patients is one of the most clinically impactful findings in perioperative cardiology of the past decade [3]. Yet the trial’s single-centre design, its predominantly open thoracotomy population, and the near-absence of replication studies [69] mean that its findings have not been translated into routine clinical practice at most centres. A multicentre replication of the PRESAGE paradigm—using standardised NT-proBNP assays, contemporary surgical approach distribution, and updated prophylactic protocols—would be the single most valuable clinical trial the field could conduct.

## 13. Conclusions

NT-proBNP is a biologically rational, mechanistically grounded, and clinically validated perioperative biomarker in lung cancer surgery. Its elevation after pulmonary resection reflects a multi-hit pathophysiological process—intraoperative RV afterload mismatch during OLV, early postoperative ischaemia–reperfusion injury and neurohormonal activation, and sustained pulmonary vascular bed reduction with wave reflection and RV-PA uncoupling—that unfolds across four temporal phases and persists, in its haemodynamic consequences, for months beyond the index procedure. The dose–response relationship between resection extent and NT-proBNP elevation is mechanistically coherent and clinically reproducible. The PRESAGE trial has established proof-of-concept that NT-proBNP-guided prophylaxis can meaningfully reduce postoperative atrial fibrillation in high-risk patients. And the emerging dual biomarker strategy combining NT-proBNP with high-sensitivity troponin offers a two-dimensional risk characterisation that exceeds the discriminative capacity of either marker alone.

Against these strengths, two limitations define the boundaries of current clinical applicability. First, no thoracic surgery-specific NT-proBNP threshold has been validated in a multicentre, prospective fashion: the 182.3 ng/L cut-off derived by Salvatici and operationalised by PRESAGE remains the most clinically actionable single value in the literature, but its generalisability to contemporary minimally invasive surgical populations is unestablished. Second, the bifurcation of the evidence base between BNP and NT-proBNP assays—without cross-calibration—prevents direct synthesis of approximately half the available threshold data and imposes a layer of numerical imprecision on all attempts at clinical translation.

The research priorities that emerge from this review are correspondingly clear. A multicentre prospective study using standardised NT-proBNP assays, stratified by resection extent and surgical approach, with predefined outcome-specific thresholds and serial perioperative sampling—including intraoperative measurements correlated with pulmonary artery pressures during OLV—would resolve the most important evidence gaps identified across all sections of this review. The OLV duration hypothesis requires direct testing through studies correlating cumulative single-lung ventilation time with serial natriuretic peptide kinetics. The PRESAGE paradigm requires replication in a contemporary multicentre cohort. And the long-term prognostic value of postoperative NT-proBNP surveillance—beyond the immediate perioperative period and into the months of pulmonary vascular remodelling that follow major resection—requires prospective evaluation with hard clinical endpoints.

NT-proBNP measurement is inexpensive, rapid, widely available, and mechanistically informative in the specific haemodynamic context of thoracic surgery. The evidence reviewed here supports its systematic integration into perioperative assessment for lung cancer resection candidates—not as a binary threshold test, but as a continuous physiological signal to be interpreted within a structured clinical framework that accounts for resection extent, surgical approach, assay platform, measurement timing, and the individual patient’s cardiac reserve.

## Figures and Tables

**Table 1:** Three-tier BNP-based risk stratification for cardiopulmonary complications after lung cancer surgery (adapted after [46]).

BNP Tier (pg/mL)	Complication Rate	Diagnostic Performance	Clinical Interpretation
<30 pg/mL Low risk	11%	Sensitivity 79% Specificity 83% AUC 0.85	Proceed with standard care Routine perioperative monitoring
30–100 pg/mL Intermediate risk	47%	Cut-off: 30 pg/mL (optimal threshold)	Enhanced monitoring Consider preoperative cardiology review
>100 pg/mL High risk	85%	4 in 5 patients will develop complications	High-level vigilance Optimise comorbidities before resection

Note: Thresholds derived from BNP assays. Application to NT-proBNP measurements requires assay-specific recalibration due to differences in clearance kinetics and circulating concentrations. AUC, area under the receiver operating characteristic curve; BNP, B-type natriuretic peptide.

**Table 2:** Identified thresholds from studies specific to thoracic surgery.

Study	Year	Biomarker	Timing	Cut-Off	Outcome	Performance	Population	Ref.
Salvatici et al.	2010	NT-proBNP	Pre- and postop	182.3 ng/L	POAF	Highest combined sensitivity + specificity (ROC-derived)	400 lung cancer surgery patients	[15]
Cardinale et al. (PRESAGE)	2016	NT-proBNP	Pre- and postop	182.3 ng/L (adopted from Salvatici)	POAF (for prophylaxis selection)	29% of patients classified high-risk; 40% AF rate in controls	1116 lung cancer surgery patients	[3]
Nojiri et al.	2010	BNP	Preoperative	30 pg/mL	POAF	Sensitivity 77%, specificity 93%, AUC 0.90	80 lung cancer patients (lobectomy/segmentectomy/wedge)	[46]
Nojiri et al.	2011	BNP	Preoperative	30 pg/mL	Cardiopulmonary complications	Sensitivity 79%, specificity 83%, AUC 0.85	Elderly lung cancer patients	[46]
Nojiri et al.	2015	BNP	Preoperative	30/100 pg/mL (3-tier)	Cardiopulmonary complications	Complication rates: 30 (11%), 30–100 (47%), >100 (85%)	Lung cancer surgery	[14]
Toufektzian et al.	2015	NT-proBNP	Preoperative	113 pg/mL	POAF	8-fold increased risk of AF	Non-cardiac thoracic surgery (best evidence review)	[49]
Cagini et al.	2014	BNP	Postop day 1	118.5 pg/mL	Cardiopulmonary complications	OR 2.94; AUC 0.654	294 pulmonary resection patients	[27]
Amar et al.	2019	BNP	Preoperative	Continuous (75th vs. 25th percentile: 57.5 vs. 12.5 pg/mL)	POAF	OR 2.08 per IQR increase; model C-statistic 0.736	635 lung/oesophageal resection patients	[8]
Bu et al.	2026	NT-proBNP	Preoperative	Continuous (incorporated in nomogram)	POAF	Model AUROC 0.718–0.753	4449 elderly VATS patients	[9]

## Data Availability

No new data were created or analyzed in this study. Data sharing is not applicable to this article.
